# Cyasterone has a protective effect on steroid-induced Osteonecrosis of the femoral head

**DOI:** 10.1371/journal.pone.0293530

**Published:** 2023-10-30

**Authors:** Youqiang Sun, Mengmeng Liang, Yuemeng Xing, Yinfan Duan, Shuangxiao Zhang, Baogui Deng, Xiaobing Xiang, Bengen Zhou

**Affiliations:** 1 Department of Sports Medicine, The First Affiliated Hospital of Guangzhou University of Chinese Medicine, Guangzhou, 510405, Guangdong Province, China; 2 Lingnan Medical Research Center of Guangzhou University of Chinese Medicine, Guangzhou, 510405, Guangdong Province, China; 3 Department of Obstetrics, Guangdong Women and Chifldren Hospital, Guangzhou, 510010, Guangdong Province, China; 4 The First Clinical College of Guangzhouf University of Chinese Medicine, Guangzhou, 510405, Guangdong Province, China; 5 Department of Sports Medicine, Heyuan Hospital of Chinese Medicine, Heyuan, 517000, Guangdong Province, China; National Research Centre, EGYPT

## Abstract

**Context:**

Cyasterone alleviated the apoptosis of BMSCs induced by Dexamethasone via the PI3K/AKT signaling pathway. In addition, Cyasterone had a protective effect on SIONFH model rats by reducing the percentage of empty bone lacunae.

**Objective:**

To study the effect of Cyasterone on apoptosis of rat BMSCs and its function on the SIONFH rat model.

**Methods:**

Rat BMSCs were cultured and divided into Control, DXM and Cyasterone (DXM+Cyasterone) groups. The apoptosis of each group was detected by flow cytometry, the expressions of Caspase-3 and Caspase-9 were detected by immunofluorescence staining, and the mRNA and protein expressions of AKT, BAX, P53, P85, Bcl-2 and Cytochrome C were detected by qPCR and WB. In animal experiments, the femoral head of rats were subjected to HE staining and Micro-CT to observe the necrosis and repair conditions.

**Results:**

The apoptosis rate of DXM and Cyasterone groups increased compared with Control group, and the apoptosis rate of Cyasterone group decreased compared with DXM group. Compared with DXM group, the mRNA expression of BAX, P53, P85 and Cytochrome C in Cyasterone group were increased, while the protein expression of AKT and Bcl-2 decreased. The histopathological and morphological analysis showed that Cyasterone promoted the trabecular bone structure in rat, which evenly benefit for the repair of SIONFH.

**Conclusion:**

Cyasterone can reduce the apoptosis of rat BMSCs induced by Dexamethasone, and help promoting the bone repair in SIONFH rats.

## 1 Introduction

Osteonecrosis of the femoral head (ONFH) is a refractory disease in orthopedics. It refers to the damage or interruption of blood supply of the femoral head, resulting in the death of bone marrow components and bone cells in the head, and then, due to the self-repair of the femoral head and structural changes in the femoral head, it finally developed into a series of collapse pathological changes and a series of clinical function disorder and pain. According to statistics every year, there are about 400,000 newly discovered ONFH patients in China, among which about 40% of ONFH patients need artificial hip replacement [1]. Although ONFH is not life-threatening, but seriously affecting the quality of life of patients. In recent years, glucocorticoids (GCs) have been widely used in clinical practice to control many serious internal diseases. However, GCs are also a double-edged sword. While treating diseases, they often bring about complications-steroid-induced osteonecrosis of femoral head (SIONFH). At present, GCs has become the main cause of ONFH disease [2].

It was reported that excess GCs increase the level of apoptosis of osteoblasts and osteocytes [3] and prolong the life of osteoclasts [4]. Bone marrow-derived mesenchymal stem cells (BMSCs) are playing pivotal roles in maintaining the dynamic bone homeostasis, which influencing the pathogenesis of bone-related diseases, such as osteoporosis and osteopetrosis [5,6]. It was shown that the low doses of GCs would induce BMSC differentiation into osteoblasts in vitro effects of GCs on BMSCs [7,8], while high doses result in apoptosis and cell death [9]. Taken into account that the new bone is mainly dependent on osteoblasts that arise from BMSCs, high doses of GCs on BMSCs is undoubtedly influence the bone loss in SIONFH [10]. Above all, excessive GCs can induce apoptosis of BMSCs, leading to the collapse of subchondral bone structure, which will finally result in the disease of SIONFH. Therefore, protect the function of BMSCs in SIONFH is essential for the clinical treatment of SIONFH.

It was reported that Dexamethasone (DXM) could induce apoptosis of osteoblasts (OB) and BMSCs by activating caspases. Caspase3 is a cysteinyl aspartate specific proteinase, which makes an important role in the apoptotic cell death [11]. On the other hand, B-cell lymphoma-2 (Bcl-2), Bcl-2-associated X (Bax), Cytochrome C, cleaved caspase3 (C-caspase3), and cleaved caspase9 (C-caspase9) proteins have also been proved to regulate the apoptosis in OB and BMSCs [12]. These proteins were highly related to the PI3K/Akt pathway.

The PI3K/Akt pathway is a signal transduction pathway involved in cell growth, survival, and proliferation [13]. In recent years, some studies reported that the PI3K/Akt pathway is closely related to the apoptosis of osteoblasts and BMSCs [14]. The inhibition of PI3K/Akt pathway may contribute to the apoptosis of BMSCs under the stimulation of GCs.

Cyasterone, one of the main extracts from the root of Achyranthes bidentata, was demonstrated of having a positive effect in the treatment of osteoporosis and osteoporotic fractures [15]. Clinical experiments show that by inhibiting osteoclast differentiation and promoting osteoblast differentiation, Cyasterone helps protect against osteoporosis caused by estrogen deficiency. Previous studies have shown that Cyasterone can stimulate in vitro migration of BMSCs in rats, accelerate fracture healing and shorten fracture healing time. In addition, Cyasterone was also demonstrated as a natural EGFR inhibitor, might be a promising anti-cancer agent [16]. However, it has not been reported in affecting the GCs-induced apoptosis of BMSCs. In this study, we evaluated whether or not Cyasterone can effectively protect BMSCs of rat after exposure to Dexamethasone in vitro and in vivo of SIONFH model, to investigate the effect of Cyasterone on Dexamethasone-induced apoptosis of BMSCs and SIONFH rats, thus to explore the mechanism and potential of Cyasterone in clinical treatment of SIONFH.

## 2 Materials and methods

### 2.1 Reagents and antibodies

Cyasterone (purity>98%), dimethylsulfoxide (DMSO), type I collagenase, Dexamethasone, formaldehyde, penicillin-streptomycin and trypsin-EDTA were acquired from Sigma Chemical Co (MO, USA). Antibodies AKT, Bcl-2, BAX, P53, P85, Caspase3, Caspase9, Cytochrome C, GADPH and secondary antibodies were obtained from Sigma Technology (MO, USA). Cell-Counting Kit-8 (CCK-8) and the RNA extraction kit were purchased from Beyotime (Shanghai, China). Thermo Fisher Scientific (Gibco, NYC, USA) supplied the Bovine serum albumin (BSA), fetal bovine serum (FBS), alpha-minimal essential medium (α-MEM), and 10% EDTA solution.

### 2.2 Cell culture and treatment

BMSCs were isolated and cultured according to the previously described methods [17], and they were characterized by flow cytometric phenotype identification using the mesenchymal stem cell criteria (Positive for CD90 and CD29, and negative for CD34 and CD45) [18]. The bone marrow was flushed out from the tibia and femur of SD rats and seeded into 75cm^2^ culture flasks with α-MEM, which containing 10% FBS and 1% penicillin-streptomycin under conditions of 5% CO^2^ and 37°C. Every 2~3 days, the medium was changed to eliminate non-adherent cells. When the adherent cells became confluent, they were removed with 0.25% Trypsin-EDTA and passaged at a ratio of 1:2. BMSCs were incubated in α-MEM medium with different concentrations of Cyasterone (0μM, 1μM, 5μM,10μM and 20μM) for 24h and 48h after being exposed to 10^-6^M Dexamethasone for 24h.

### 2.3 Cell viability

BMSCs were treated with different concentrations of Cyasterone (0μM, 1μM, 5μM,10μM, 20μM) for 48h to test the toxicity of Cyasterone. The Cell Counting Kit-8 was used to test cell viability according to the manufacturer’s instructions, and the absorbance of each well was measured at 450nm using a microplate reader.

### 2.4 Flow detection of apoptosis

1×10 ^6^~3×10 ^6^ BMSCs were collected, centrifuged with 1mL PBS at 1500rpm for 3 mins, and washed twice. Use double distilled water to dilute 5×Binding Buffer into 1×Binding Buffer. The cells were collected and resuspended in 300μL of precooled 1×Binding Buffer. After adding 3μL Annexin V-FITC and 5μL PI to each tube and slight mixing, the samples were incubated for 10 min at room temperature in the dark. Subsequently, 200μL of precooled 1×Binding Buffer was added to each tube, mixed evenly, and detected by an upflow meter for assessing cell apoptosis. The Annexin V-FITC conjugates were detected with the FL1 channel of the FACS Calibur flow cytometer, and the PI was read on the FL2 channel, which was analyzed by the Cell Quest software (BD Biosciences).

### 2.5 Immunofluorescence

Cells were fixed with 4% paraformaldehyde for 15min, and then 0.5%TritonX-100 was added for 20min at room temperature. Then 5%BSA was added to the petri dish and sealed at 37°C for 30min. Then, a sufficient amount of diluted primary antibody Caspase-3 (1:100) or Caspase-9 (1:100) was added into the petri dish after incubated overnight at 4°C. After that, diluted fluorescent secondary antibody was added and incubated at 37°C for 30min. DAPI was added and incubated for 5min in the dark, and then the specimens were nucleus stained. Petri dishes were sealed with 50% glycerin, and images were observed and collected under a confocal microscope (CKX53, OLYMPUS, Tokyo, Japan).

### 2.6 PCR

RNA was extracted from cells in each group according to the instruction of the RNA extraction kit. cDNA was synthesized according to the reverse transcription kit. The cDNA was used as template for detection on the fluorescence quantitative PCR instrument. Primers were self-designed as follows in Table 1.

**Table 1:** Primers of AKT, Bax, P53, P85, BCL-2, Cytochrome C and β-actin.

The primers	Primer sequences	Length (nt)	The length of the product (bp)	The annealing temperature (°C)
AKT1 F	TAGGCATCCCTTCCTTACAGC	21	114	60.52
AKT1 R	CGCTCACGAGACAGGTGGA	19		
Bax F	TGGCGATGAACTGGACAACA	20	125	60.48
Bax R	CCCAGTTGAAGTTGCCGTCT	20		
P53 F	ACAGTTAGGGGGTACCTGGC	20	118	60.78
P53 R	AGCTCGATGCTCATATCCGAC	21		
P85 F	ACAAAGCCGAGAACCTATTGC	21	108	59.64
P85 R	TGACTTCGCCATCTACCACTAC	22		
BCL-2 F	GGACGCGAAGTGCTATTGGT	20	141	60.52
BCL-2 R	AGTATCCCACTCGTAGCCCC	20		
Cytochrome C F	CTTGGGCTAGAGAGCGGGA	19	132	61.45
Cytochrome C R	GTGGCACTGGGCACACTTTT	20		
β-actin F	GCCATGTACGTAGCCATCCA	20	375	59.5
β-actin R	GAACCGCTCATTGCCGATAG	20		

Note: The primers were designed from the pubmed website https://www.ncbi.nlm.nih.gov/nuccore/?term= and were synthesized by the company of General Biosystems (Anhui) Co., Ltd.

### 2.7 Western blot

Each group (The Control group, DXM group and DXM+Cyasterone group) of cells was added into the corresponding lysate (RIPA), and the cells were lysed at 4°C for 30min. The supernatant was centrifuged at 10000rpm/min for 10min, and the total protein was obtained by carefully absorbing the supernatant. Protein concentration was determined by BCA kit. Protein denaturation, loading, electrophoresis for 1–2h, and wet film transfer for 30–50min. The primary antibody solution was incubated overnight at 4°C. The secondary antibodies were incubated at room temperature for 1–2h. Drop ECL exposure solution on the membrane, exposure. The gray values of antibody bands were analyzed by "Image J" software. The antibodies including β-actin (1:1000), p53 (1:1000), AKT (1:1000), BAX (1:1000), Cytochrome C (1:1000), p85 (1:1000), Bcl-2 (1:1000) and secondary antibodies (1:2000).

### 2.8 Animals and Animal Model of SIONFH

30 Sprague Dawley (SD) rats were adaptively reared for 1 week, and randomly divided into 3 groups: the Cyasterone group (n = 10), model group (n = 10), control group (n = 10). The animal was provided by the Laboratory Animal Center of Guangzhou University of Traditional Chinese Medicine (ethical number: SCX-2018-0034). All methods were performed in accordance with the ARRIVE guidelines and relevant regulations. The method of SIONFH model was injecting Lipopolysaccharide (LPS) (20ug/kg) into the abdominal cavity alternately on both sides for every 24 hours of 2 times. In the third day, the bilateral gluteal muscles were alternately injected with 6α-Methylprednisolone (MPS) 40 mg/kg for 3 times, with an interval of 24 hours each time. Control group: Perform the same operation as above, and inject the corresponding volume of normal saline. Experimental group: The rats were allowed to recover for 3 days after the modeling model finished, and the Cyasterone was injected intraperitoneally once every other day for 6 weeks, a total of 21 times, and with a volume of 100ul each time. At the 16th week, the animals were euthanized with an overdose (150mg/kg) of pentobarbital administered intraperitoneally. The femoral heads were collected for Micro-CT and HE staining analysis.

### 2.9 Histological analysis

The femoral head samples were fixed in 4% paraformaldehyde at 4°C for 24 h, then transferred to a 10% EDTA solution for decalcification for 4 weeks. After dehydrating and embedding the samples in paraffin blocks, 5-μm sections were cut and underwent the HE staining procedure. Finally, they were observed under a microscope (OLYMPUS, BX53, Japan) to identify the empty lacunae, pyknotic nuclei of osteocytes, and broken bone trabeculae. We analyzed the proportion of empty lacunae in the femoral head samples according to established criteria [19].

### 2.10 Micro-CT analysis

The specimens were scanned on a Micro-CT system (Skyscan1172 Bruker Belgium). Micro-CT parameters and indicators: The scanning voltage is 80 kV, the electric current is 500μA, and the scanning resolution is 10.44μm. After scanning, mimics software was used for 3D reconstruction with voxels of 1024μm×1024μm×1024μm [20]. The detection parameters include tissue volume (TV, Tissue Volume), bone volume (BV, Bone Volume); the Connection Density (Connectivity Density, Conn.D.), Structure Model Index (SMI), Trabecular Number (Tb.N), Trabecular Thickness (Tb.Th), Trabecular Separation (Trabecular Number Separation/Spacing, Tb.Sp); and 3D reconstruction. The region of interest (ROI) was 8mm.

### 2.11 Statistical analysis

All data were statistically analyzed using SPSS19.0 (Chicago, IL, USA), and quantitative results were expressed as mean ± standard deviation (X±S). The data were analyzed by one-way analysis of variance with a post-hoc test to determine the significance among the different groups, P values < 0.05 were considered significant.

## 3 Results

### 3.1 Cyasterone inhibits DXM-induced cytotoxicity in BMSCs

**Fig 1:**
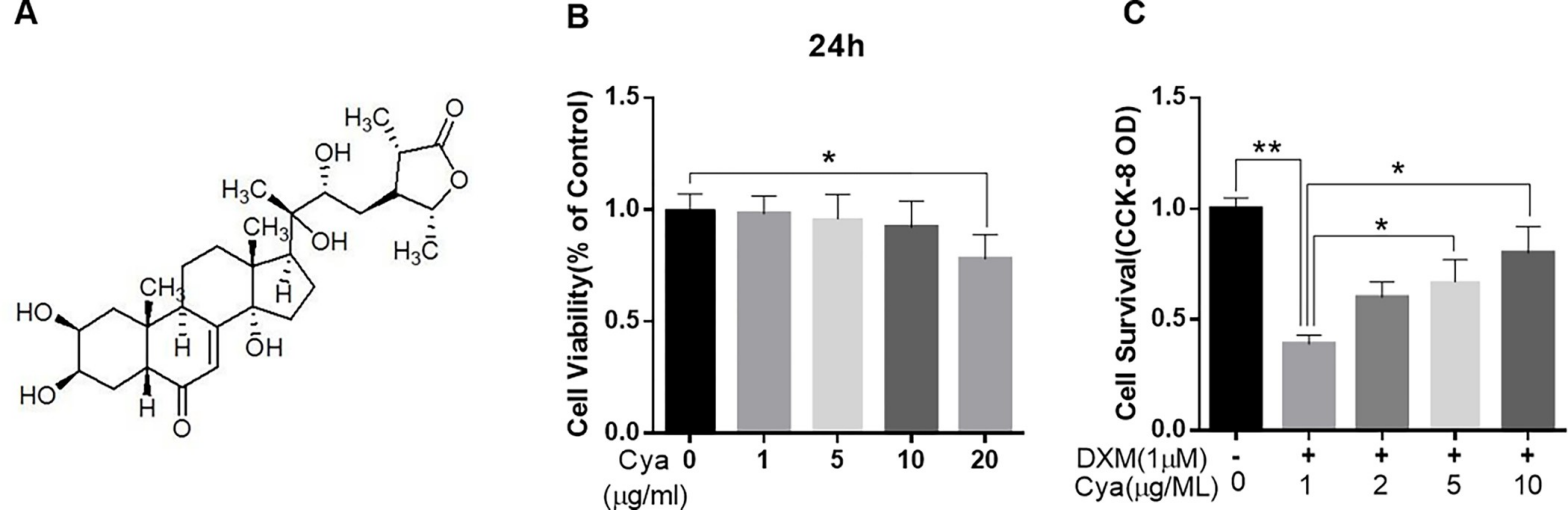
Potential Cytotoxicity of Cyasterone on BMSCs. The chemical structure of Cyasterone (A). The cytotoxic effects of Cyasterone on BMSCs were determined by increasing concentrations (0, 1, 5, 10 and 20μM) for 24 hours using a CCK8 assay (B, C). Cyasterone was not cytotoxic to BMSCs at concentrations of 1 to 10μM at 24h. Cyasterone ameliorate DXM-induced cell death in a concentration-dependent manner, especially when the concentration of Cyasterone at a level of 10μM. (Values represent the averages±S.D. Significant differences between different groups are indicated as *P < 0.05, **P < 0.01, vs DXM alone treatment group, n = 3).

The chemical structure of Cyasterone is shown in Fig 1A. BMSCs were cultured with Cyasterone at different concentrations (0, 1, 5, 10, and 20μM). As showed in Fig 1B, Cyasterone was not cytotoxic to BMSCs at concentrations of 1 to 10μM at 24h. And treatment of BMSCs with different concentrations of Cyasterone could significantly reduce the DXM-induced cell death in a concentration-dependent manner, especially when the concentration of Cyasterone at a level of 10μM (Fig 1C).

### 3.2 The result of flow detection of apoptosis rate in BMSCs

**Fig 2:**
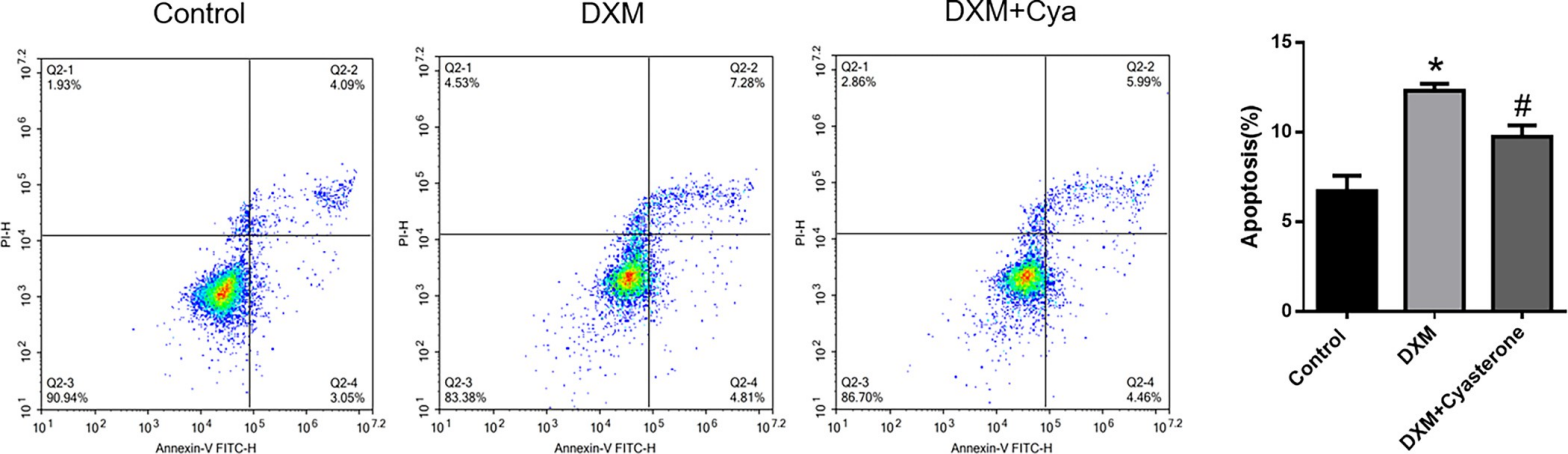
The apoptosis rate of BMSCs in each group. The apoptosis rate of DXM group and DXM+Cyasterone group was higher than that of the Control group, while the apoptosis rate of DXM+Cyasterone group was lower than that of the DXM group. It was showed that Cyasterone reduced the DXM-induced apoptosis compared with DXM group. (*P<0.05, vs Control, #P<0.05, DXM+Cyasterone vs DXM, n = 3).

The apoptosis rate of in vitro cultured groups of BMSCs was detected by flow cytometry, which was showed in Fig 2. The apoptosis rate of DXM group and DXM+Cyasterone group was higher than that of the Control group, while the apoptosis rate of DXM+Cyasterone group was lower than that of the DXM group. The specific apoptosis rate in the Control group, DXM group and DXM+Cyasterone group were 6.72±1.48, 12.32±0.68 and 9.74±1.10 respectively. Obviously, Cyasterone reduced the DXM-induced apoptosis compared with DXM group.

### 3.3 Immunofluorescence detection results of Caspase-3 and Caspase-9 expression in cells

**Fig 3:**
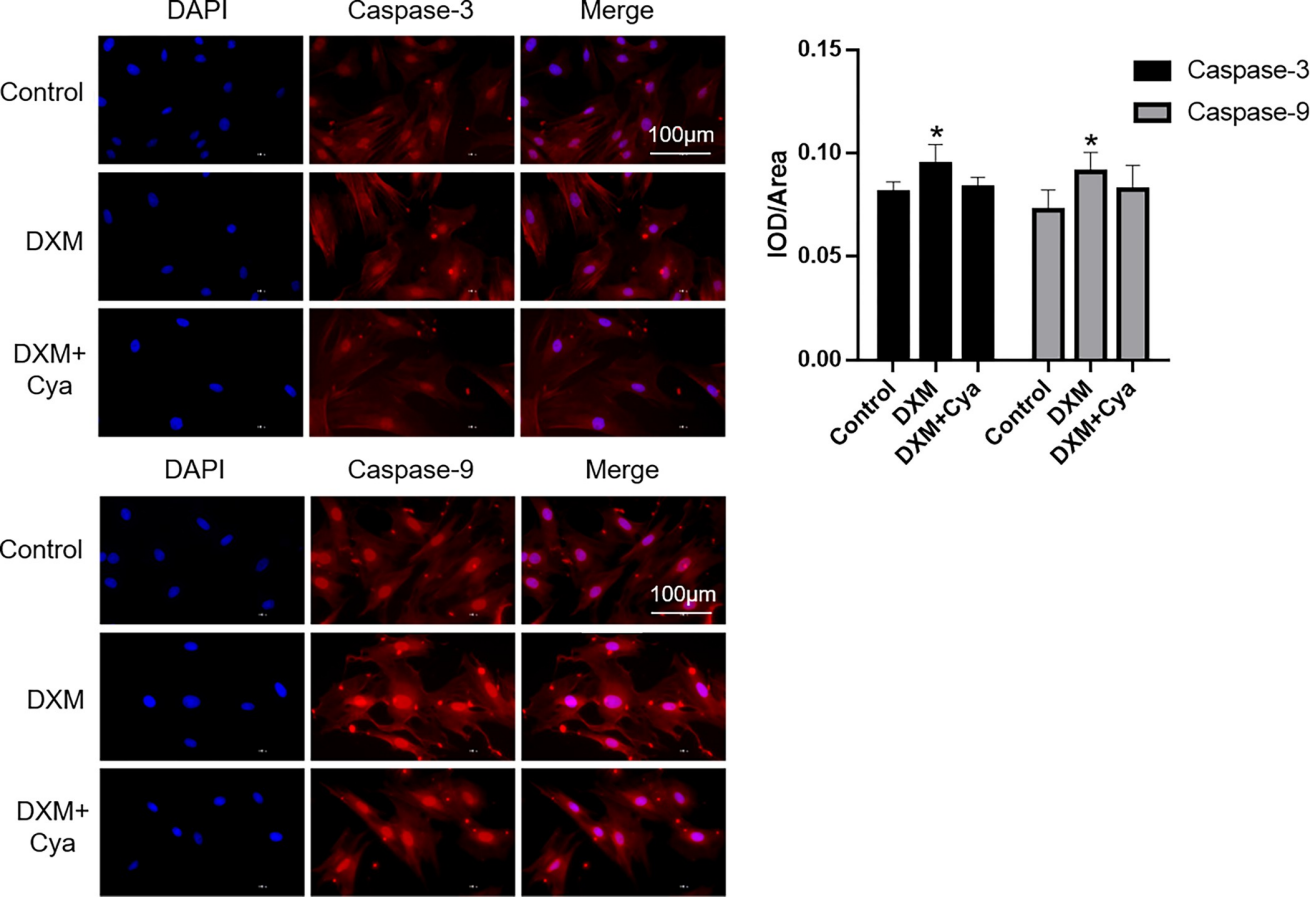
Results of immunofluorescence detection of the expression of Caspase-3 and Caspase-9 in cells. The bar graph showed both caspase-3 and caspase-9 were punctually overexpressed in the cytoplasm. (400×, the nucleus is stained in blue, and the target gene protein is stained in red, Cya is short for Cyasterone, P<0.05, DXM+Cya vs DXM, n = 3).

The results of immunofluorescence detection of Caspase-3 and Caspase-9 expressions in vitro cultured groups of BMSCs were shown in Fig 3. Both caspase-3 and caspase-9 were punctually overexpressed in the cytoplasm.

### 3.4 The expression of AKT, BAX, P53, P85, Bcl-2, Cytochrome C mRNA and protein in cells

**Fig 4:**
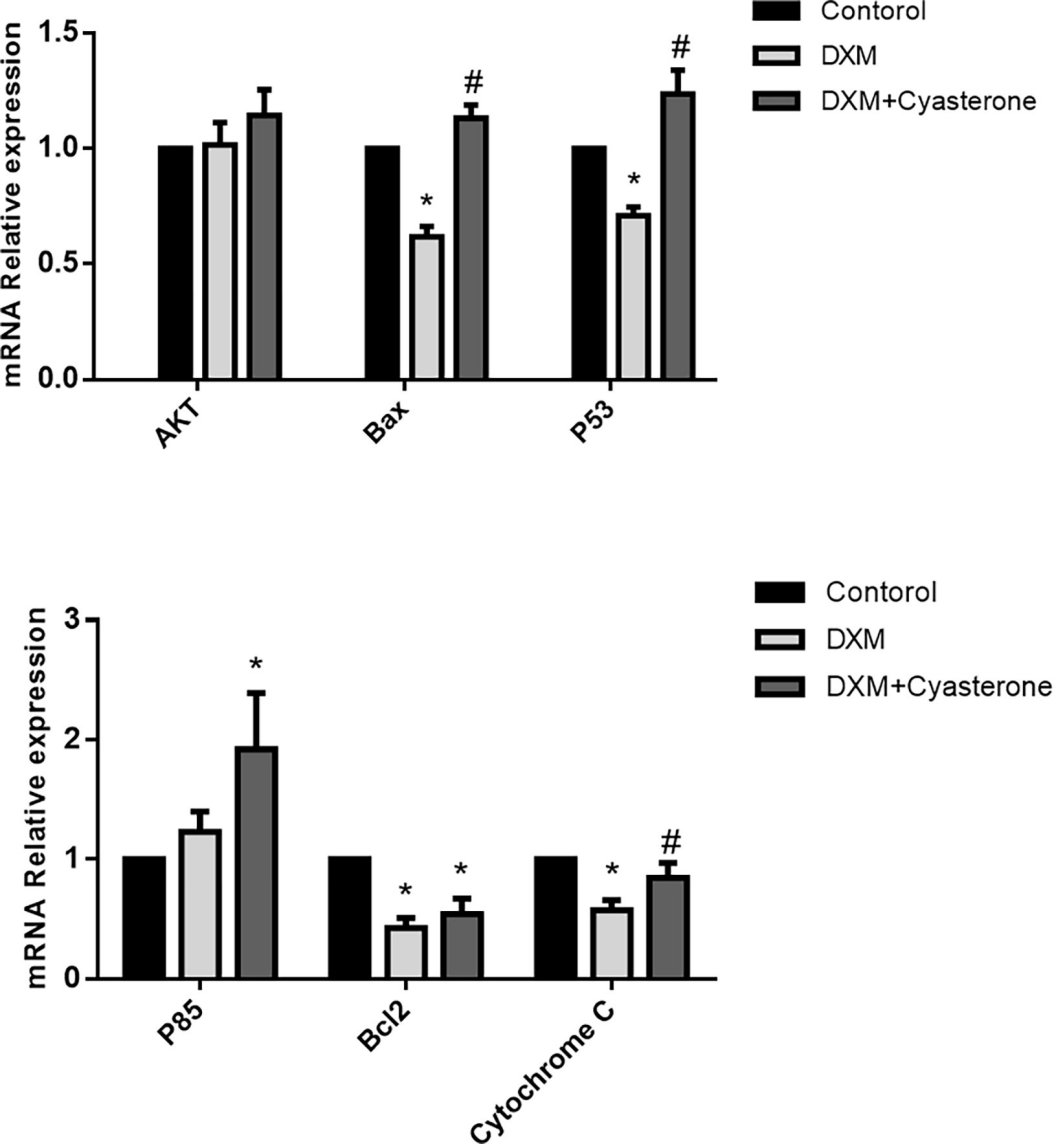
Results of PCR of the mRNA expression of AKT, BAX, P53, P85, Bcl-2, Cytochrome C in cells. The mRNA expression of BAX, P53, Bcl-2 and Cytochrome C decreased in DXM group compared with the Control group, but the mRNA expression of BAX, P53 and P85 increased in DXM+Cyasterone group compared with that DXM group. (*P<0.05, vs Control; #P<0.05, DXM+Cyasterone vs DXM, n = 3).

**Fig 5:**
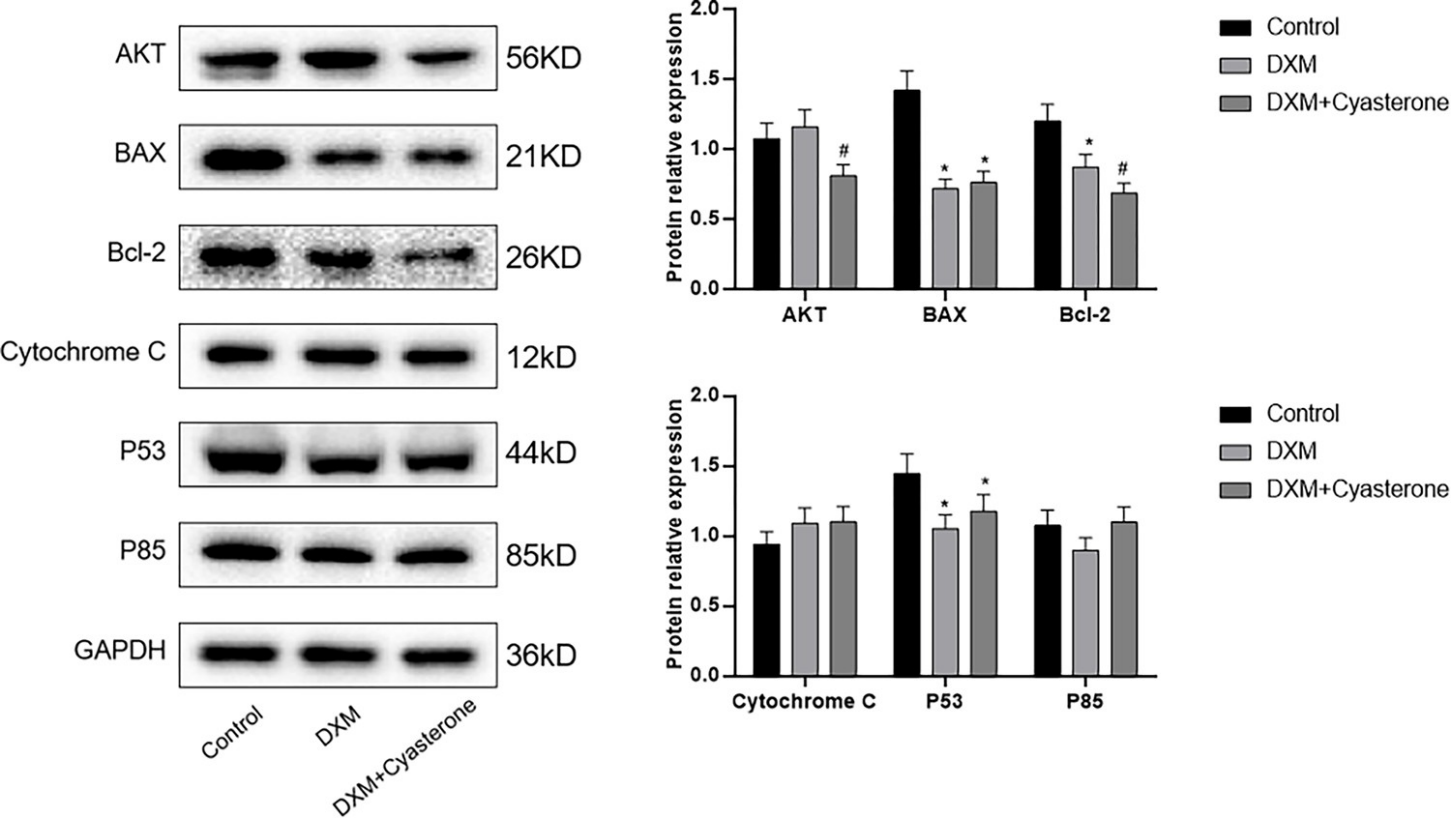
Results of WB of the protein expression of AKT, BAX, P53, P85, Bcl-2, Cytochrome C in cells. The protein expression level of BAX, Bcl-2 and P53 decreased in DXM group compared with Control group, while the protein expression level of AKT, Bcl-2 decreased in DXM+Cyasterone group compared with DXM group. (*P<0.05, vs Control; #P<0.05, DXM+Cyasterone vs DXM, n = 3).

The results of PCR of AKT, BAX, P53, P85, Bcl-2 and Cytochrome C mRNA expression in vitro cultured groups of BMSCs were shown in Fig 4. The WB results of protein expression are shown in Fig 5. There was no significant difference in AKT mRNA expression between groups, but protein expression decreased in DXM+Cyasterone group. The mRNA expression of BAX decreased in DXM group. Protein expression decreased in DXM group and DXM+Cyasterone group. The expression of Bcl-2 mRNA and protein decreased in DXM group and DXM+Cyasterone group. The mRNA expression of P53 decreased in DXM group. Protein expression decreased in DXM group and DXM+Cyasterone group. The mRNA expression of P85 increased in DXM+Cyasterone, but there was no significant difference in protein expression between groups. The mRNA expression of Cytochrome C decreased in DXM group and DXM+Cyasterone group, but there was no significant difference in protein expression between groups. In all, Compared with DXM group, the mRNA expression of BAX, P53, P85 and Cytochrome C in DXM+Cyasterone group were increased, while the protein expression of AKT and Bcl-2 decreased.

### 3.5 Results of animal experiments

After the HE staining and analysis, 18 of the 20 femoral heads in the Cyasterone group (n = 10) and the model group (n = 10) showed signs of necrosis, and the necrosis rate was 90%, with a survival rate of 100%.

**Fig 6:**
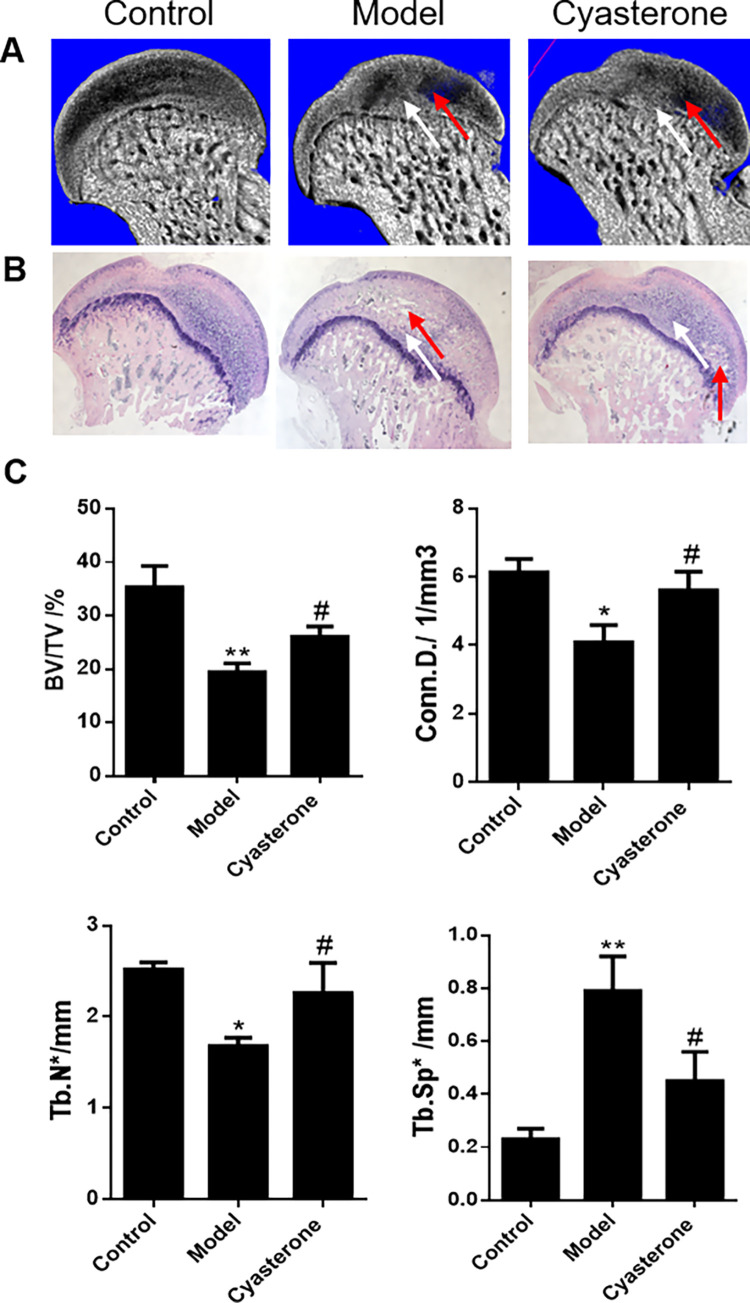
Representative images of Micro-CT and HE staining, and the structural parameters of Micro-CT. Fig 6 (A, B) showed the HE staining and Micro-CT reconstruction images of the three groups of rats; Fig 6A showed the results of Micro-CT after sampling of the femoral head of the three groups of rats; Fig 6B showed that the trabecular bone in the model group was obviously sparser and partially broken than in the Control group (Scale bar: 400 μm, the red arrow represents the necrotic area while the white arrow shows the normal area); The Fig 6C showed that the values of BV/TV, Conn.D and Tb.N increased in the Cyasterone group and the value of Tb.Sp decreased in the Cyasterone group. (The samples conformed to normal distribution and the variance was homogeneous, and one-way analysis of variance was used for comparison among multiple groups. *P<0.05, **P<0.01, Model group compared with Control group; #P<0.05, N = 5, Cyasterone group compared with model group).

The histological analysis showed in the Fig 6 is the representative pictures of HE staining of the femoral head sections of rats in the model group, Control group and Cyasterone group, respectively. Compared with the Control group, the model group were seen a wrinkle of cartilage surface, trabecular bone was sparse and obvious, fractured trabecular bone appeared in some areas, and a large number of empty bone lacuna appeared, accompanied by the accumulation of bone marrow adipocytes, and osteocyte nucleus pyknosis was seen in the bone trabeculae. The trabecular bone structure of the rats in the Control group was regular, the osteocytes in the trabecular were clearly visible, and there was no obvious empty bone lacuna, there were more hematopoietic cells in the bone marrow, and the content of fat cells was relatively small and morphological normal. In the Cyasterone group, the trabecular bone was sparse, but the shape was acceptable, and the osteoblasts were active. After Image J quantitative penalty analysis, the figure shows that the rate of empty bone lacuna in the model group was (18.14±1.15) %, the Control group was (5.57±0.97) %, and the Cyasterone group was (8.15±0.96) %, as shown in Fig 6B; The BV/TV, Conn.D, Tb.N, Tb.Sp values of the three groups were showed in the Fig 6C, the values of BV/TV, Conn.D and Tb.N increased in the Cyasterone group and the value of Tb.Sp decreased in the Cyasterone group.

## 4 Discussion

Although SIONFH pathogenesis still remains uncertain, several factors have been postulated to play pivotal roles in SIONFH [21–23], such as the abnormal differentiation of bone marrow mesenchymal stem cells and osteoblasts, the apoptosis of bone marrow mesenchymal stem cells, osteoblasts and osteoclasts, the lipid metabolism disorders, and the coagulation pathway disorders.

The apoptosis hypothesis is one of the most important theories of SIONFH. The core of the hypothesis is that the apoptosis of BMSCs and Osteoblast Cells may eventually develop into ONFH. Previous studies on the apoptosis of SIONFH mainly focused on the apoptosis of osteocytes and osteoblasts, while few studies focused on the apoptosis of BMSCs. This study would carry out researches from the perspective of apoptosis of BMSCs.

It was shown that several major signaling pathways involved in cell apoptosis, they were PI3K/AKT, Wnt/β-catenin, TGF-β/Smad signaling pathway, etc [24–26]. Among them, the most relevant pathway was the PI3K/AK signaling pathway. Previous studies have shown that DXM can induce osteoblast apoptosis and result in a SIONFH disease via PI3K/AKT pathways [27]. Thus, it may be possible to prevent SIONFH by the inhibition of osteoblast or BMSCs apoptosis.

PI3K/AKT signaling pathway, also known as PKB signaling pathway, is one of the classical autophagy signaling pathways, and it is the only known inhibitory pathway in the process of cell autophagy regulation and apoptosis. After activation, it can inhibit autophagy and protect cells from apoptosis. On the contrary, inhibition of this pathway can induce autophagy and apoptosis. Hormone-acting receptors can be found in OB, osteoclasts (OC), chondrocyte and osteocytes. These receptors can bind GCs and stimulate cell apoptosis by activating relevant signaling pathways.

As the PI3K/AKT signaling pathway plays an important role in inhibiting the apoptosis of OB and BMSCs, the inhibition of PI3K/AKT signaling pathway may lead to apoptosis of BMSCs under the stimulation of GCs.

SIONFH caused by the use of DXM and other GCs has become an urgent problem to be solved. It was reported that the mechanism of SIONFH may be related to some pathological states, such as microvascular coagulation, apoptosis, intraosseous hypertension, intravascular fat embolism, osteoporosis and arterial vascular injury [28]. In recent years, the mechanism of apoptosis has attracted our attention. Long-term and excessive use of steroid will cause changes in the microenvironment of OB and BMSCs in the femoral head, and it will result in the over-expression of apoptosis-related factors, such as BCL-2, Bax, cytochrome C, C-caspase3 and C-caspase9 [13]. These apoptotic factors give rise to cell apoptosis and tissue destruction through a series of reaction. At present, there is no effective drug in the treatment of SIONFH, and most of them end up with artificial hip replacement, so it is imperative to discover a drug which can protect and slow down the progression of SIONFH.

The above literature shows that apoptosis plays an important role in the occurrence and development of SIONFH. OB and osteocyte apoptosis have been shown to increase the occurrence of ONFH. However, there have been few studies on the apoptosis of ONFH BMSCs. Based on this literature survey, it is necessary to continue to study the mechanism of ONFH in the effect of PI3K/AKT signaling pathway on the apoptosis of BMSCs.

Therefore, from the point of view of BMSCs apoptosis, this study aims to the marker genes and proteins (Akt, bax, p53, p85, bcl-2, cytochrome c, caspase-3, caspase-9) in PI3K/AKT signaling pathway, so as to study the protective function of the Cyasterone in apoptosis of BMSC cells induced by Dexamethasone and reveal its related mechanism of preventing the progression of SIONFH.

Cyasterone has been previously reported to be a therapeutic compound for MSCs mobilisation and osteogenesis. What’s more, it could accelerate the fracture healing of the bone. However, the antiapoptotic effects of Cyasterone in SIONFH have not yet been reported by far. In our study, we demonstrated that Cyasterone plays an anti-apoptosis role through the PI3K/AKT pathways in BMSCs.

In the study, we found Cyasterone was not cytotoxic to BMSCs at concentrations of 1 to 10μM at 24h. Cyasterone ameliorated DXM-induced cell death in a concentration-dependent manner, especially when the concentration of Cyasterone at a level of 10μM. In addition, the cell apoptosis rate flow cytometry test showed that the apoptosis rate of DXM group and DXM+Cyasterone group was higher than that of Control group, and the apoptosis rate of DXM+Cyasterone group was lower than that of DXM group, which means the Cyasterone prevent the apoptosis of induced by the DXM. What’s more, the results of immunofluorescence detection of the expression of Caspase-3 and Caspase-9 in cells saw both highly expressed in cytoplasm.

In the PCR and WB experiments, we found the mRNA expressions of BAX, P53, P85 and Cytochrome C were increased, while the protein expressions of AKT and Bcl-2 were decreased, the results showed even though the DXM induced the apoptosis in BMSCs, Cyasterone have a protective effect in the gene and protein in the PI3K/AKT pathway in DXM-induced BMSCs. However, the PCR and WB results were not that matching very well, it might be the expression of gene and protein were not always the same in different stages of DXM-induced BMSCs.

In the vivo animal model of SIONFH experiment, we found that all the 20 of 20 SD rats in model and Cyasterone groups were survived, the survival rate was 100%. It shows that the modeling method of LPS (20μg/kg) ×2+MPS (40mg/kg) ×3 has high success rate and low mortality rate, which is a feasible modeling method for SIONFH.

In the SIONFH model evaluation stage, none of the 30 SD rats died, and the survival rate was 100%. The histological HE staining showed that the necrosis rate was 90% in all the animal model and Cyasterone group. After Image J quantitative penalty analysis, it shows that the Cyasterone might help reduce the rate of empty bone lacuna.

The Micro-CT analysis showed the values of BV/TV, Conn.D, Tb.N and Tb.Sp in the Cyasterone group were increased than that model group, and the difference was statistically significant (P<0.05). According to the comprehensive histopathological and morphological analysis, we concluded that Cyasterone can promote the trabecular bone structure in rat, which evenly benefit for the repair of SIONFH.

Previous studies found that the natural compound-Cyasterone have a protective effect in osteoporosis and osteoporotic fractures [15], but not in any study of the treatment of SIONFH. In addition, the early study seldom focuses on the SIONFH treatment by using a DXM-induced BMSCs cell model. In our study, we did the experiment of cell model of SIONFH by using the DXM-induced BMSCs. According to our data, we found that Cyasterone might inhibit the activation of apoptosis induced by DXM via the PI3K/AKT signaling pathway in BMSCs and it might have a protective effect on the SIONFH rat by reducing the percentage of empty bone lacunae.

## 5. Conclusions

In conclusion, this study demonstrated that Cyasterone might inhibit the activation of apoptosis in DXM-induced BMSCs via the PI3K/AKT signaling pathway. Further, Cyasterone also has a protective effect on SIONFH model rats. Taken together, these findings indicate that Cyasterone may have a therapeutic potential in the treatment of SIONFH.

## Supporting information

S1 Checklist (PDF)

S1 Fig The timeline and design of the experimental. (TIF)

S1 File (ZIP)
