# Supplementary figures and images for: Cyasterone has a protective effect on steroid-induced Osteonecrosis of the femoral head
Source: PLoS One. 2023 Oct 30;18(10):e0293530. doi: 10.1371/journal.pone.0293530 (PMC10615314; doi:10.1371/journal.pone.0293530)

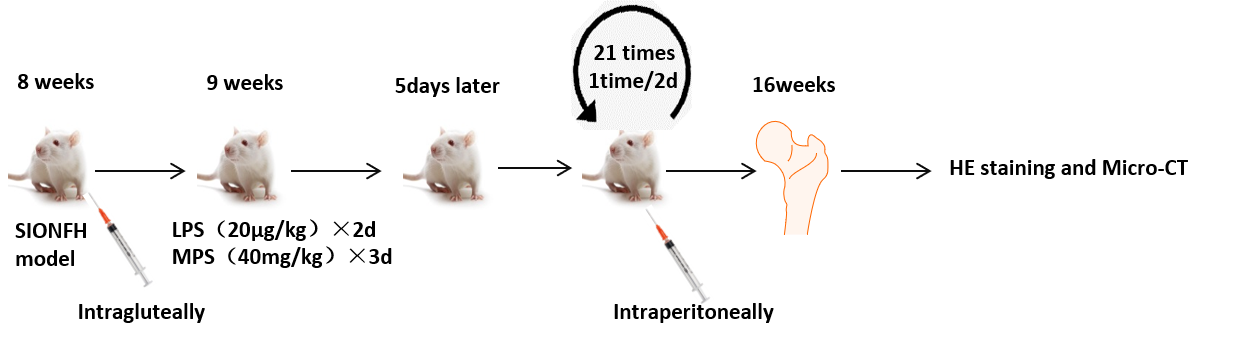

Supplement: S1 Fig — (TIF) [file pone.0293530.s002.tif]

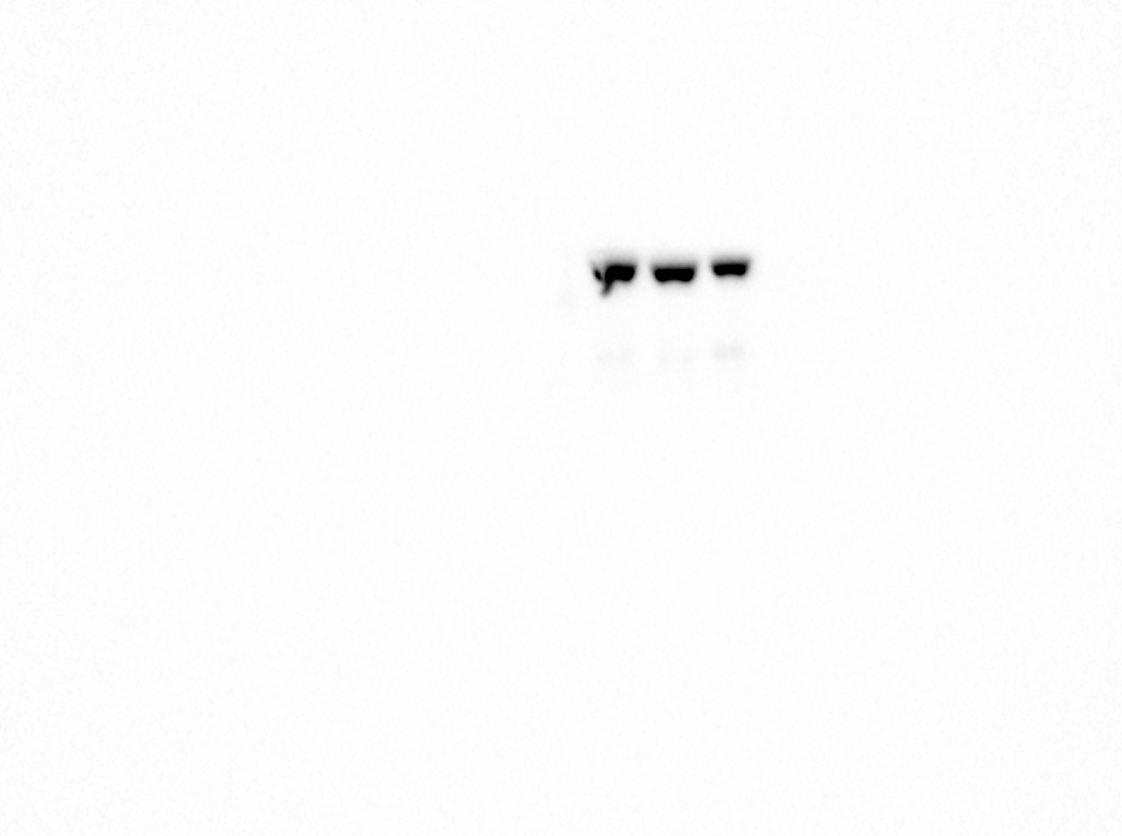

Supplement: S1 File — (ZIP) [file pone.0293530.s003.zip › Uncropped western blots/WB-akt/Administrator 2020-08-12 16 ╩▒ 39 ╖╓_Exposure_1.0sec.scn50.tif]

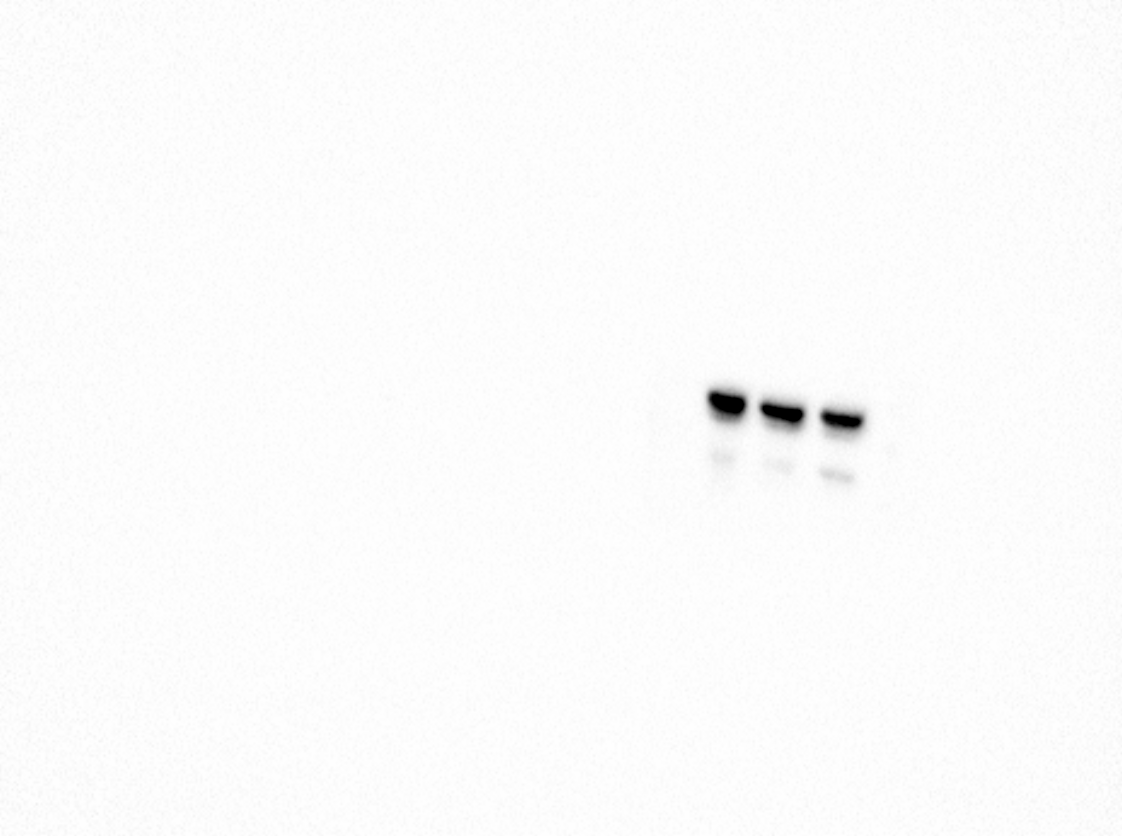

Supplement: S1 File — (ZIP) [file pone.0293530.s003.zip › Uncropped western blots/WB-akt/Administrator 2020-08-18 16 ╩▒ 19 ╖╓_Exposure_2.0sec.scn 13.tif]

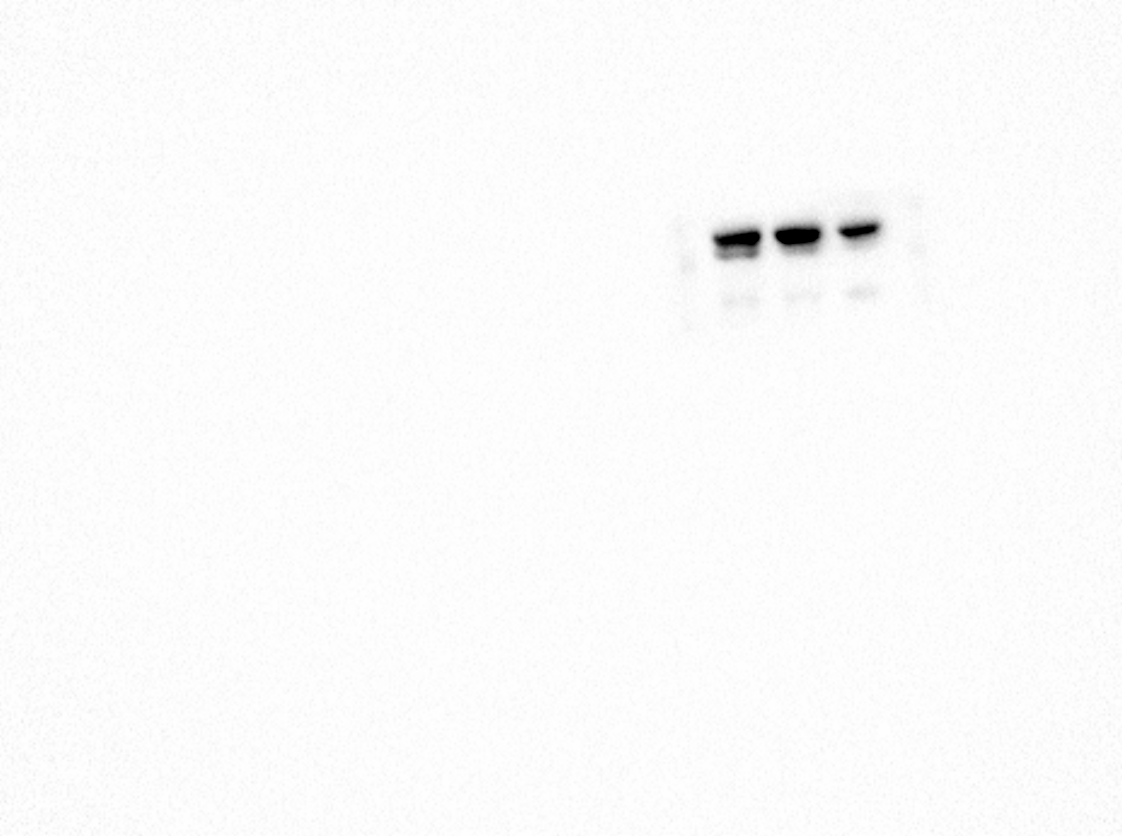

Supplement: S1 File — (ZIP) [file pone.0293530.s003.zip › Uncropped western blots/WB-akt/Administrator 2020-08-18 16 ╩▒ 20 ╖╓_Exposure_1.0sec.scn 14.tif]

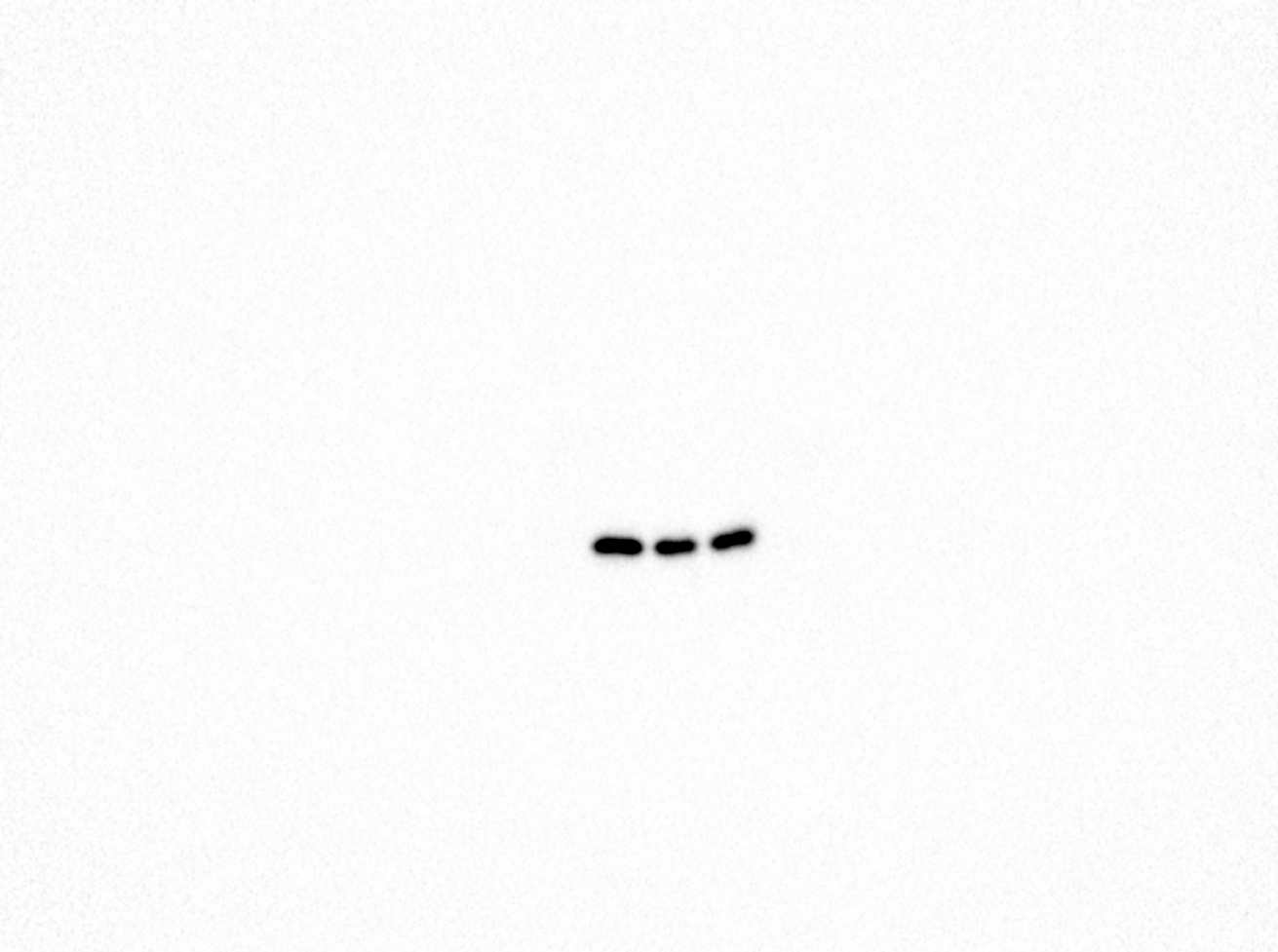

Supplement: S1 File — (ZIP) [file pone.0293530.s003.zip › Uncropped western blots/WB-bax/Administrator 2020-08-04 15 ╩▒ 18 ╖╓_Exposure_1.0sec.tif]

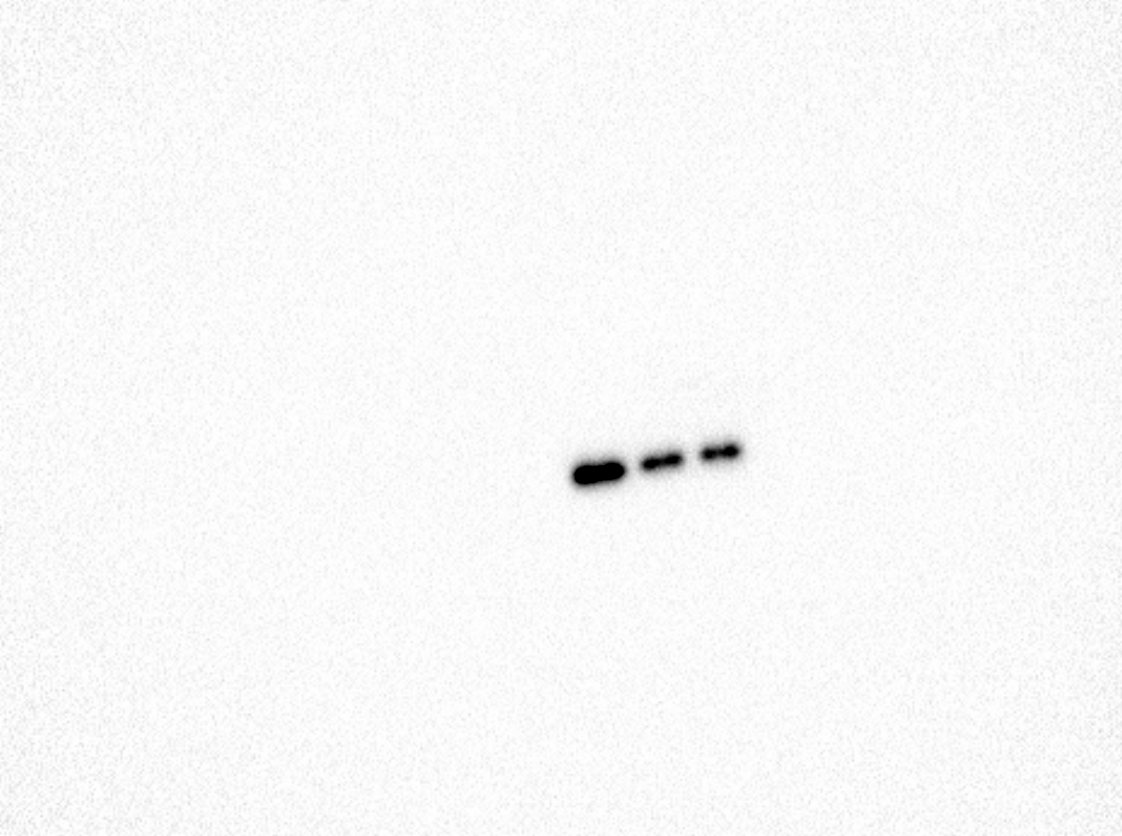

Supplement: S1 File — (ZIP) [file pone.0293530.s003.zip › Uncropped western blots/WB-bax/Administrator 2020-08-07 15 ╩▒ 25 ╖╓_Exposure_2.0sec.scn56.tif]

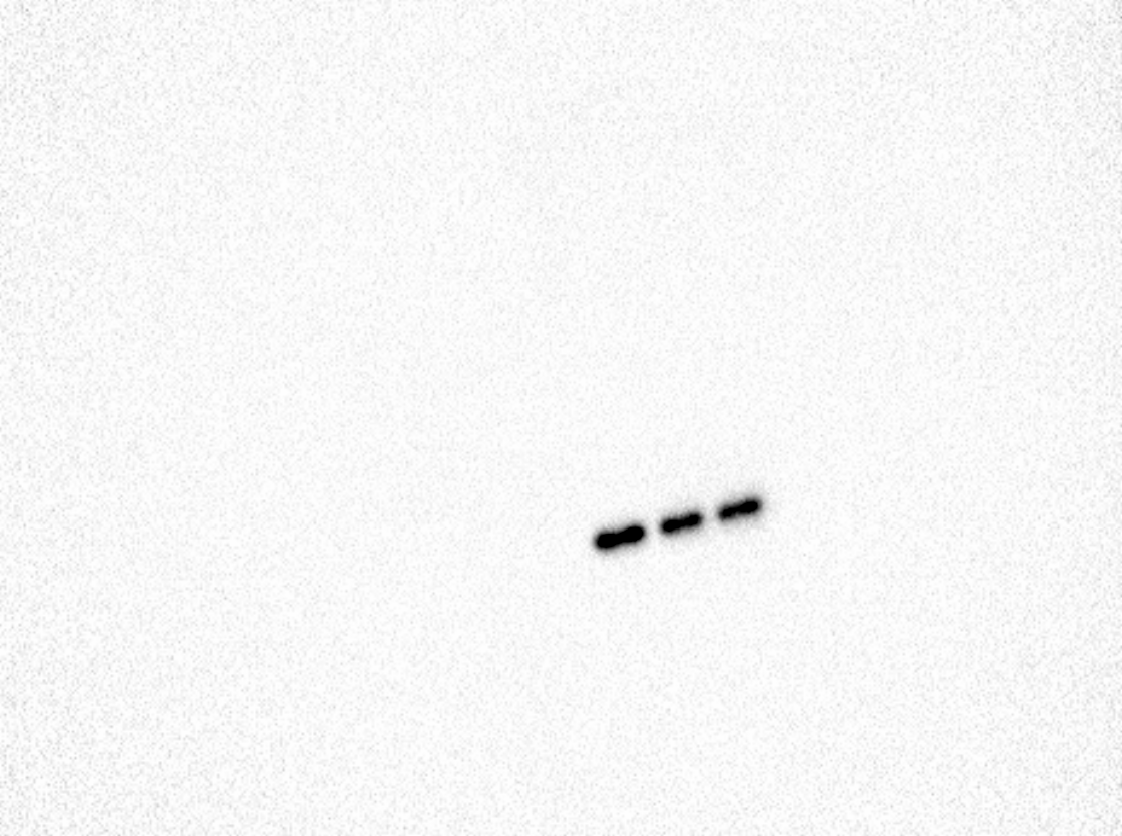

Supplement: S1 File — (ZIP) [file pone.0293530.s003.zip › Uncropped western blots/WB-bax/Administrator 2020-08-07 15 ╩▒ 25 ╖╓_Exposure_2.0sec.scn57.tif]

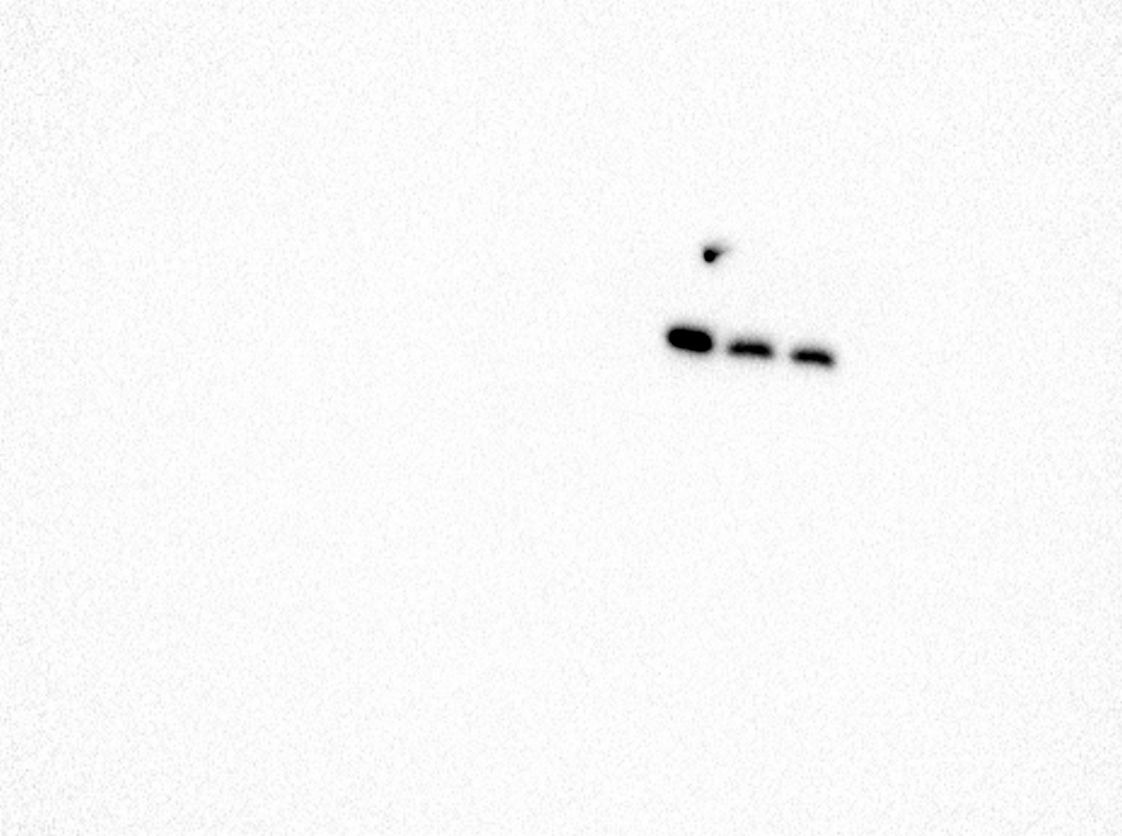

Supplement: S1 File — (ZIP) [file pone.0293530.s003.zip › Uncropped western blots/WB-bax/Administrator 2020-08-08 18 ╩▒ 03 ╖╓_Exposure_1.5sec.tif]

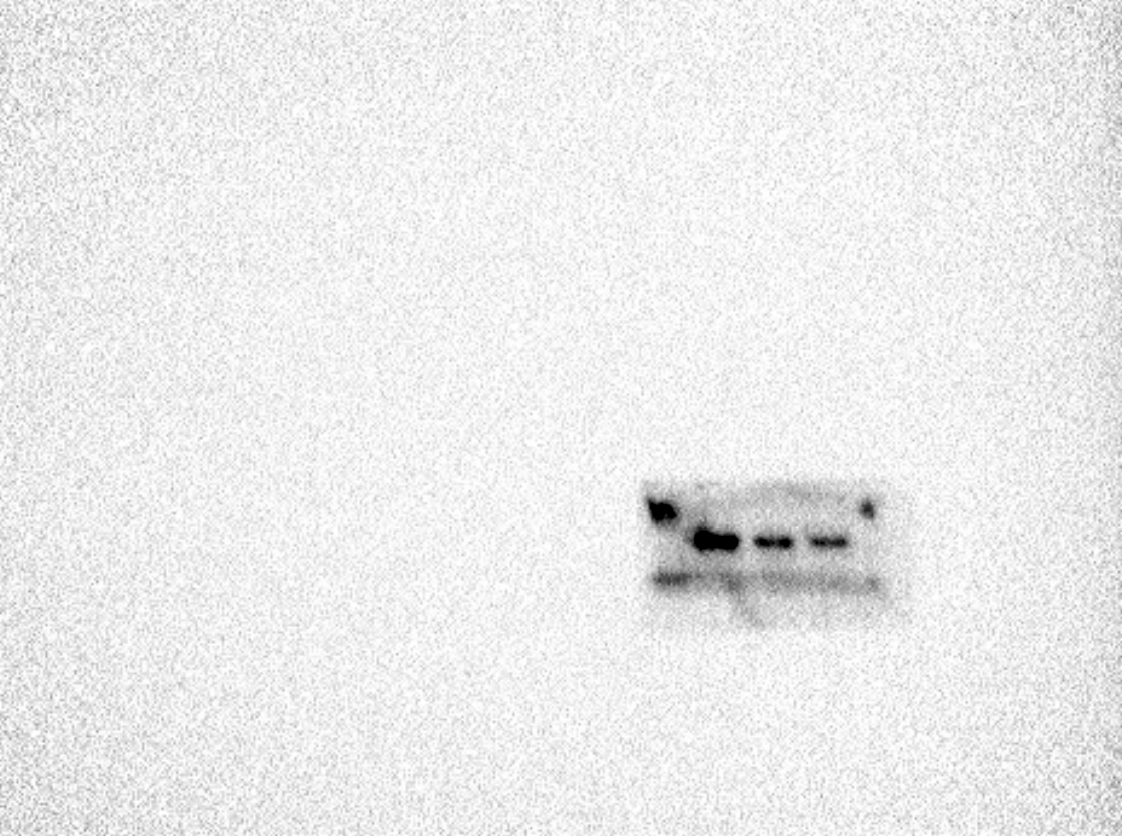

Supplement: S1 File — (ZIP) [file pone.0293530.s003.zip › Uncropped western blots/WB-bax/Administrator 2020-08-16 15 ╩▒ 38 ╖╓_Exposure_7.9sec.scn5.tif]

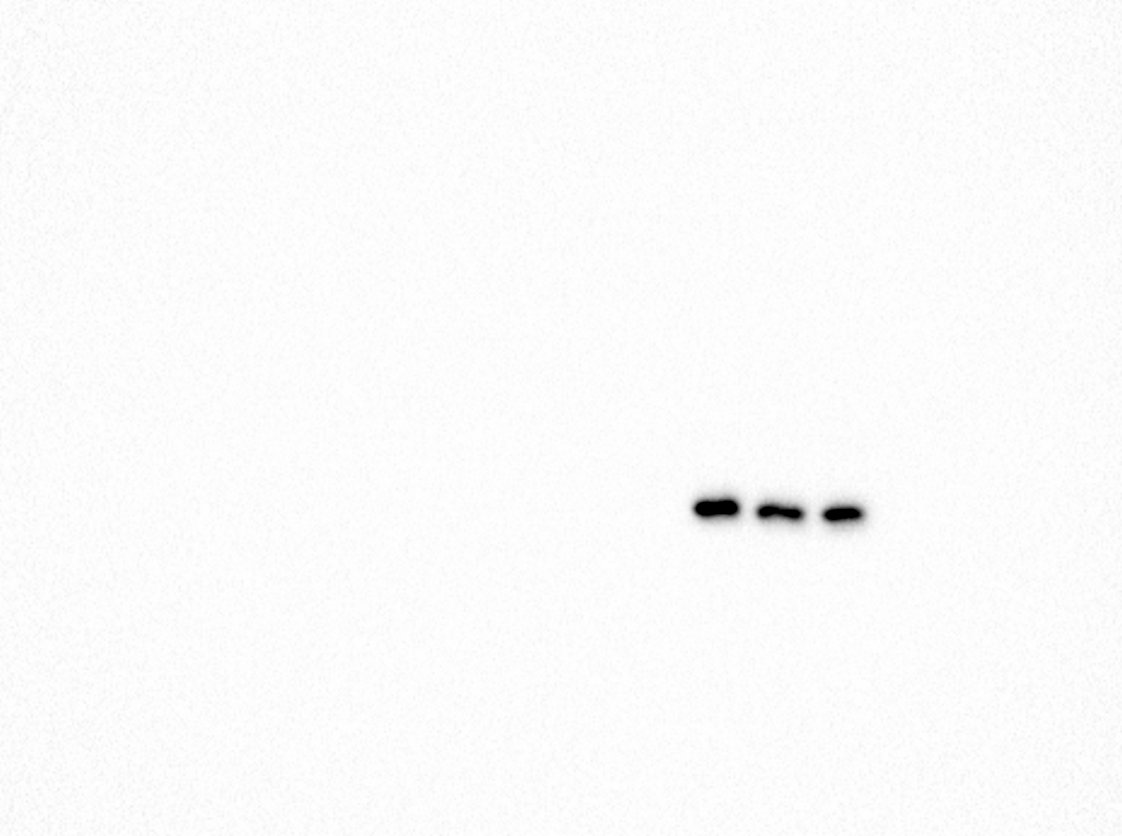

Supplement: S1 File — (ZIP) [file pone.0293530.s003.zip › Uncropped western blots/WB-bax/Administrator 2020-08-18 16 ╩▒ 50 ╖╓_Exposure_1.5sec.scn 12.tif]

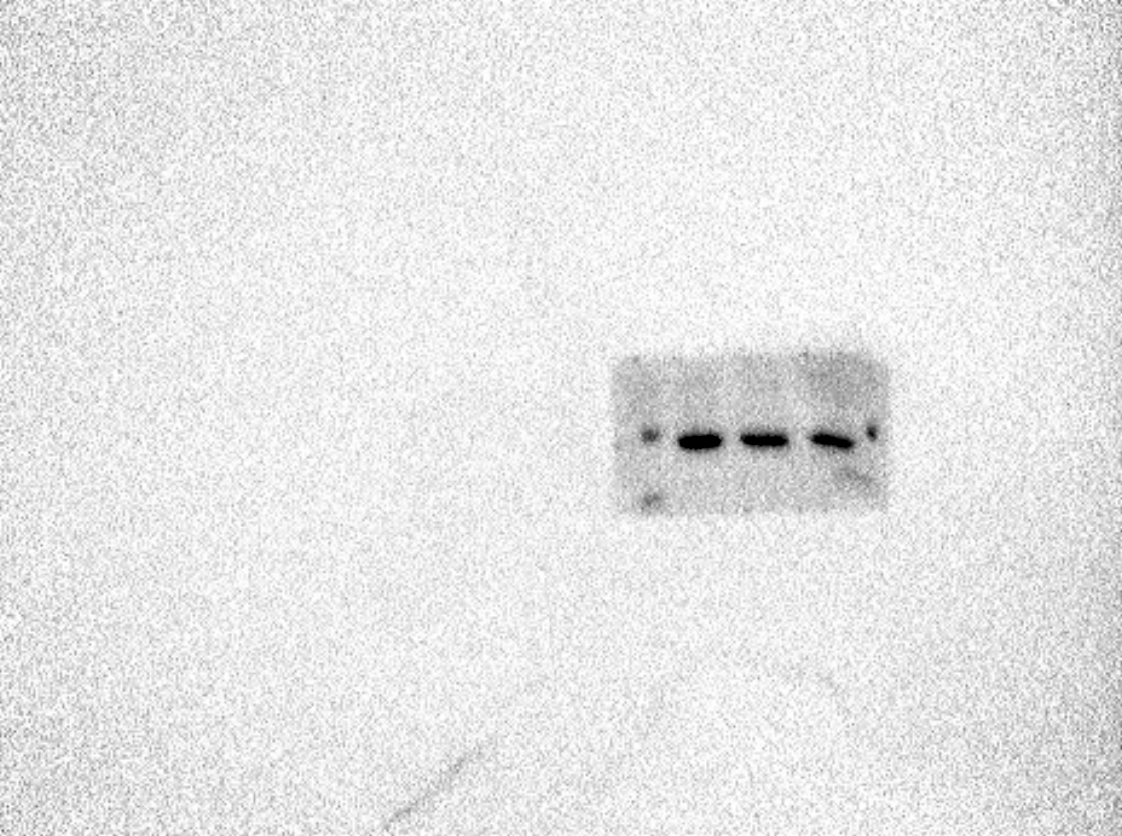

Supplement: S1 File — (ZIP) [file pone.0293530.s003.zip › Uncropped western blots/WB-bcl2/Administrator 2020-08-15 17 ╩▒ 31 ╖╓_Exposure_16.0sec.tif]

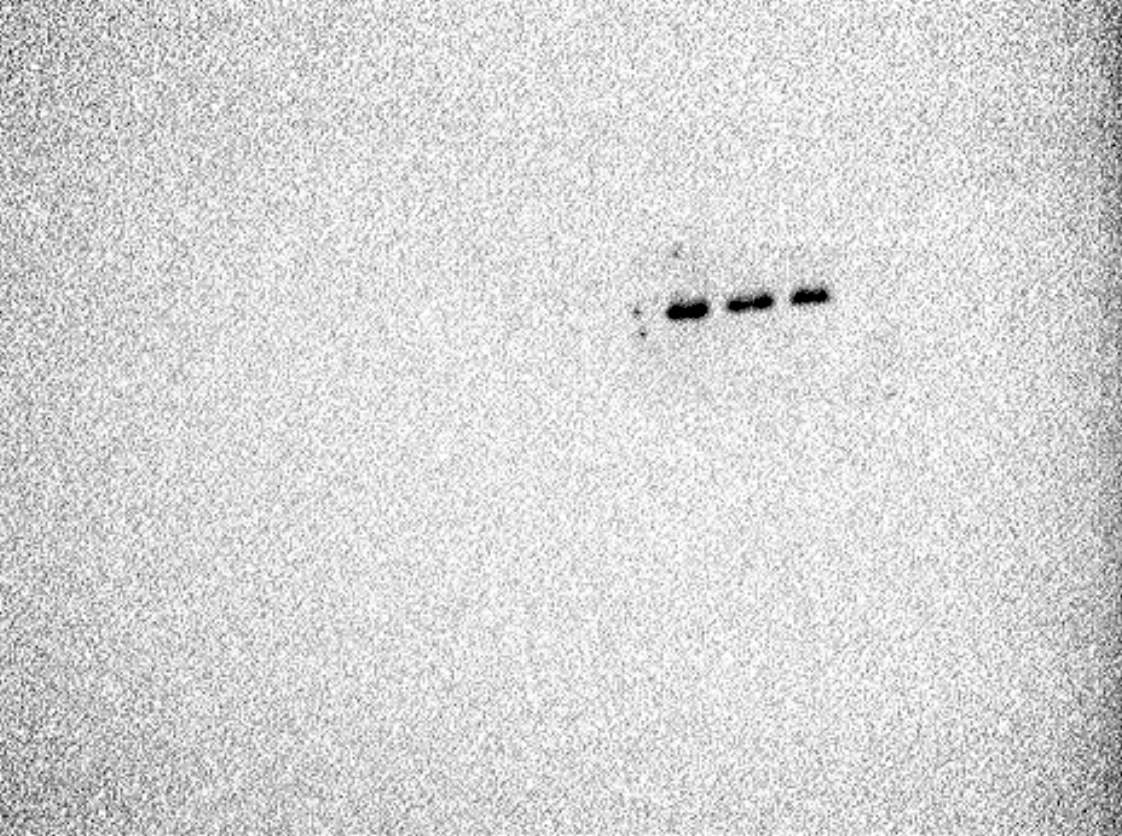

Supplement: S1 File — (ZIP) [file pone.0293530.s003.zip › Uncropped western blots/WB-bcl2/Administrator 2020-08-20 16 ╩▒ 54 ╖╓_Exposure_17.3sec.scn6.tif]

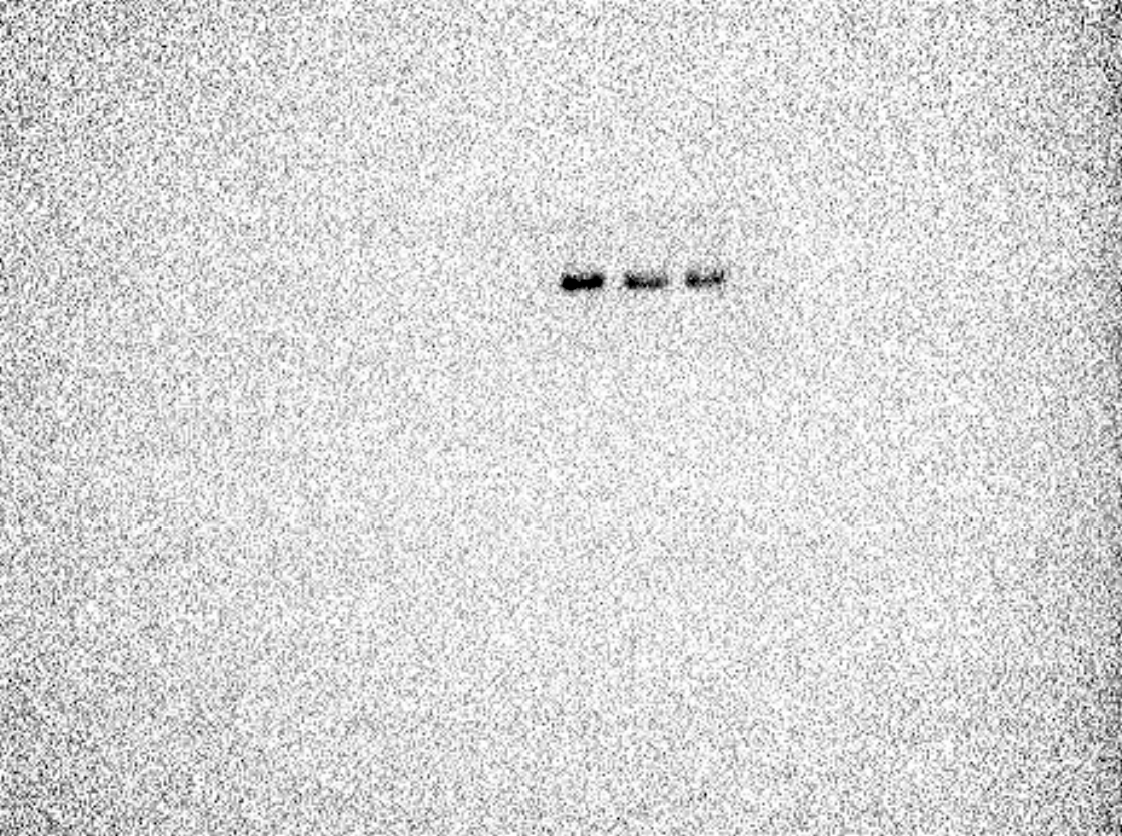

Supplement: S1 File — (ZIP) [file pone.0293530.s003.zip › Uncropped western blots/WB-bcl2/Administrator 2020-08-20 16 ╩▒ 54 ╖╓_Exposure_5.0sec.scn5.tif]

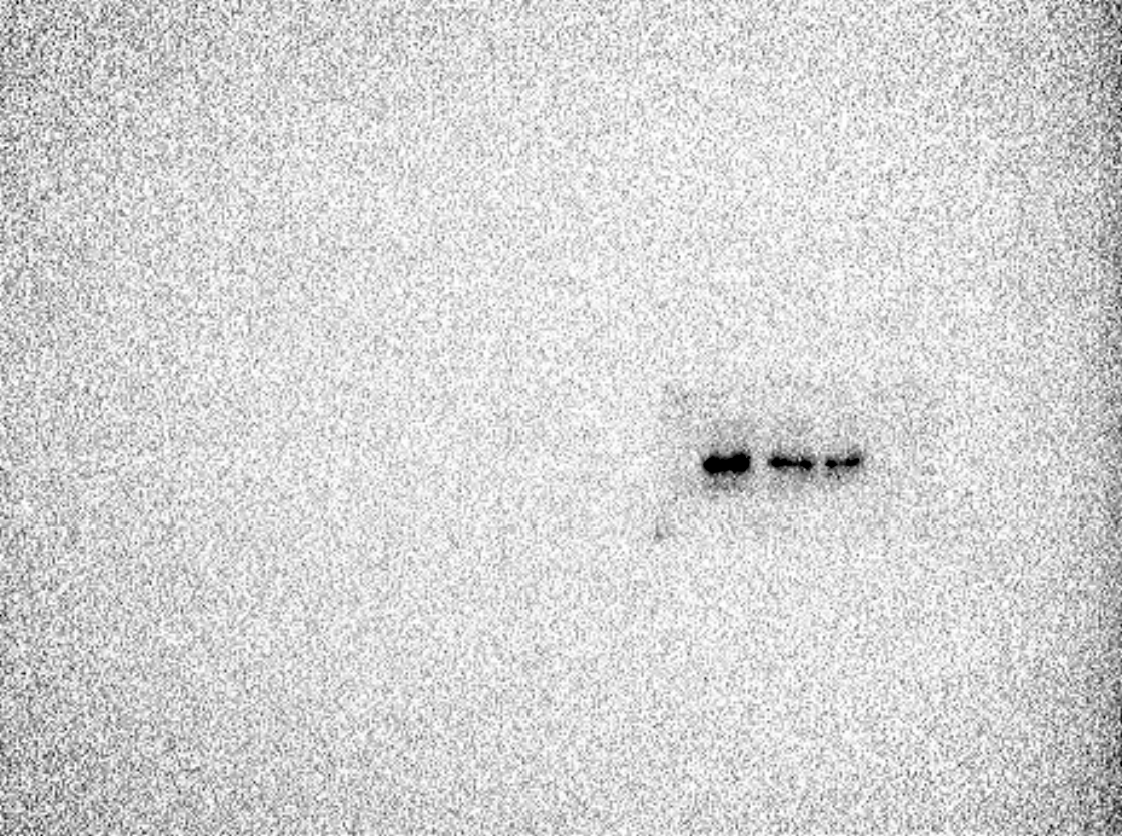

Supplement: S1 File — (ZIP) [file pone.0293530.s003.zip › Uncropped western blots/WB-bcl2/Administrator 2020-08-21 16 ╩▒ 03 ╖╓_Exposure_11.9sec.scn52.tif]

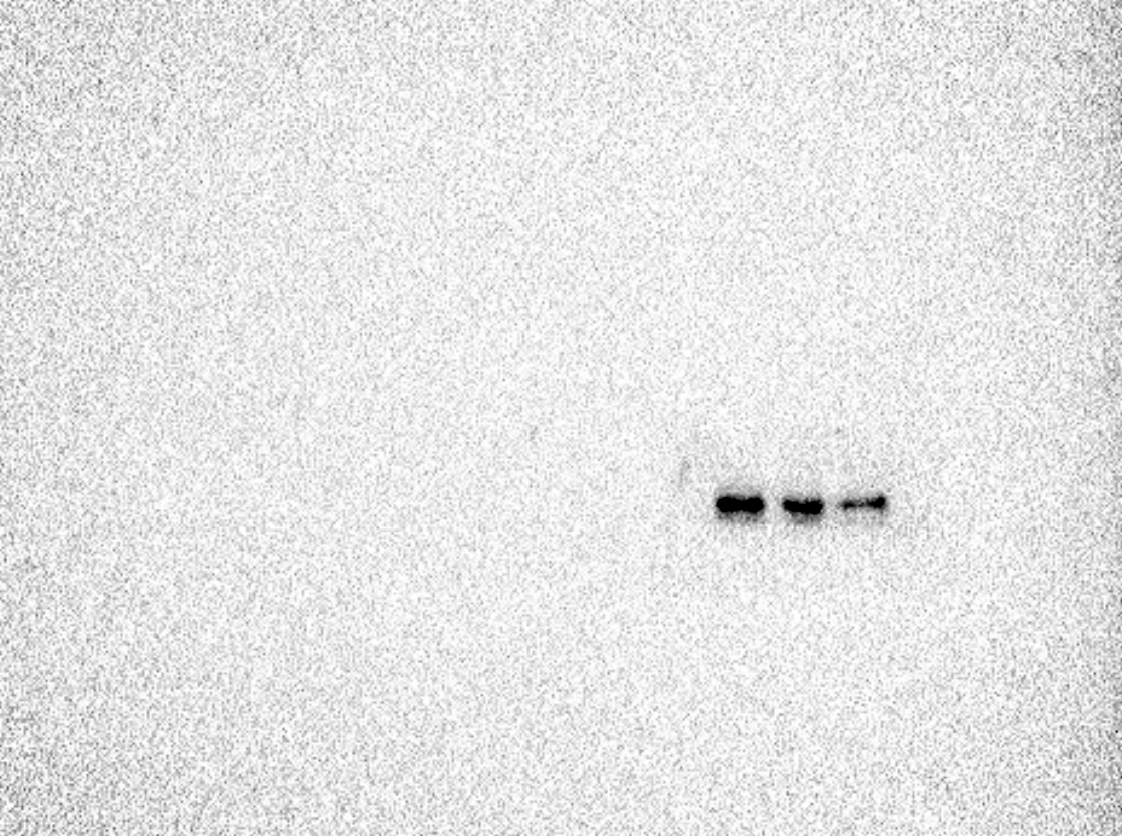

Supplement: S1 File — (ZIP) [file pone.0293530.s003.zip › Uncropped western blots/WB-bcl2/Administrator 2020-08-21 16 ╩▒ 03 ╖╓_Exposure_5.4sec.scn51.tif]

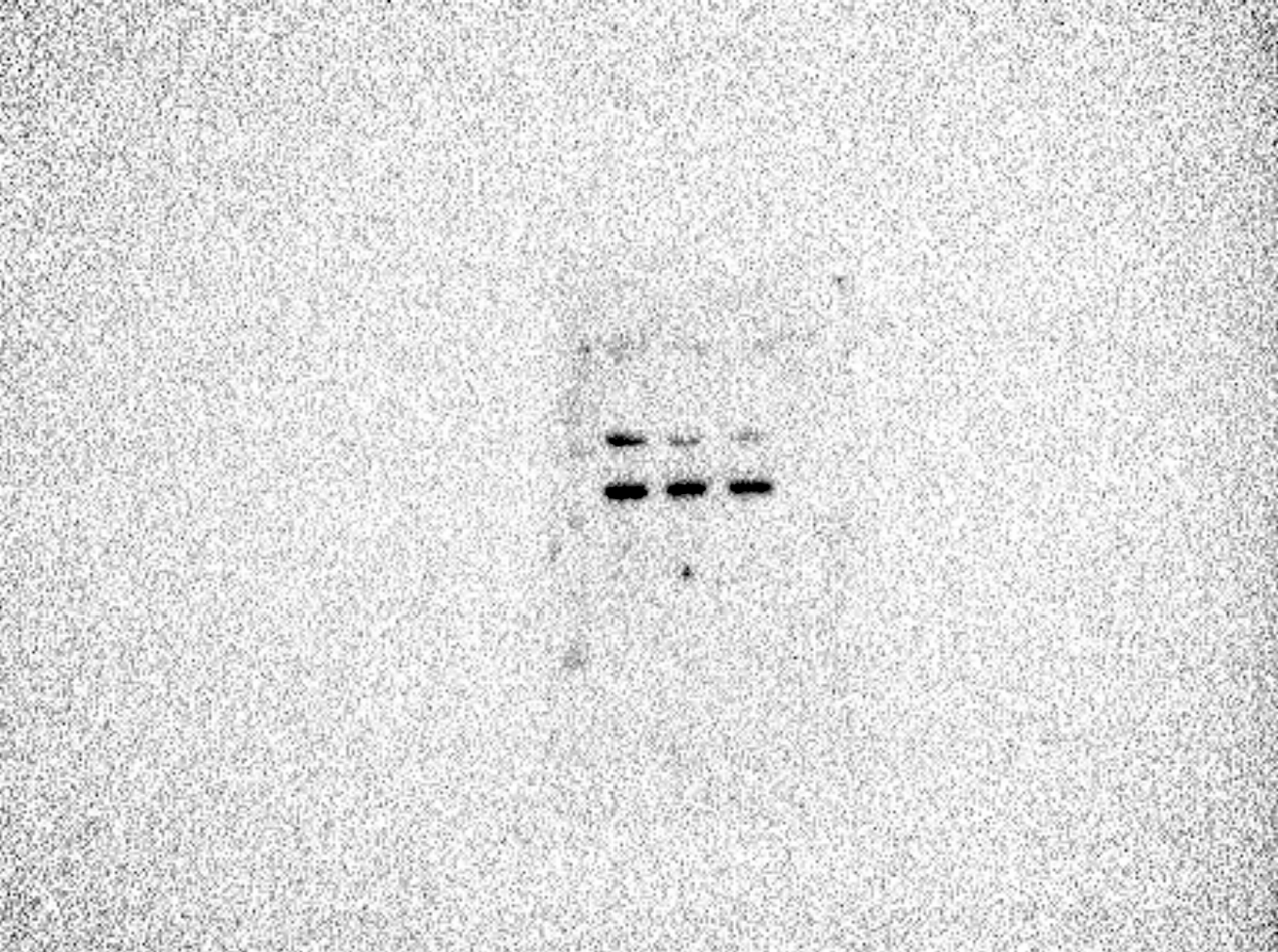

Supplement: S1 File — (ZIP) [file pone.0293530.s003.zip › Uncropped western blots/WB-Cytc/Administrator 2020-08-04 14 ╩▒ 56 ╖╓_Exposure_15.1sec.tif]

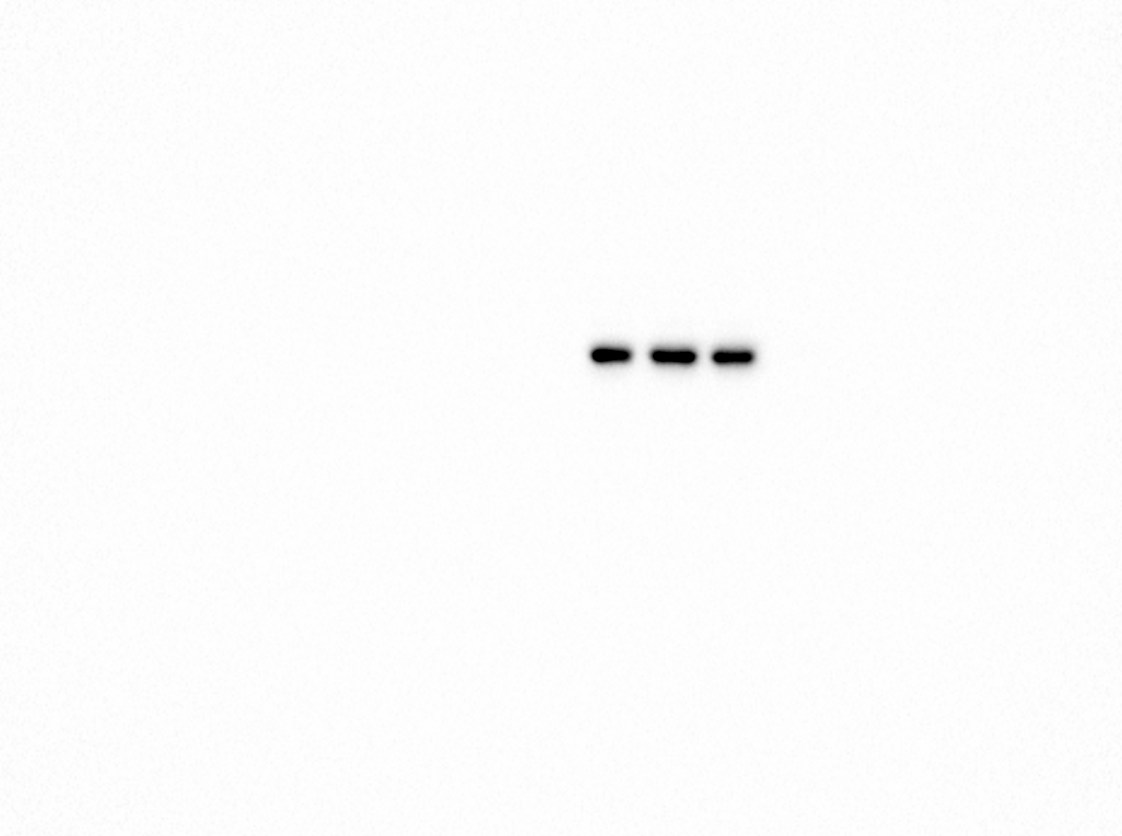

Supplement: S1 File — (ZIP) [file pone.0293530.s003.zip › Uncropped western blots/WB-Cytc/Administrator 2020-08-13 15 ╩▒ 00 ╖╓_Exposure_1.0sec.tif]

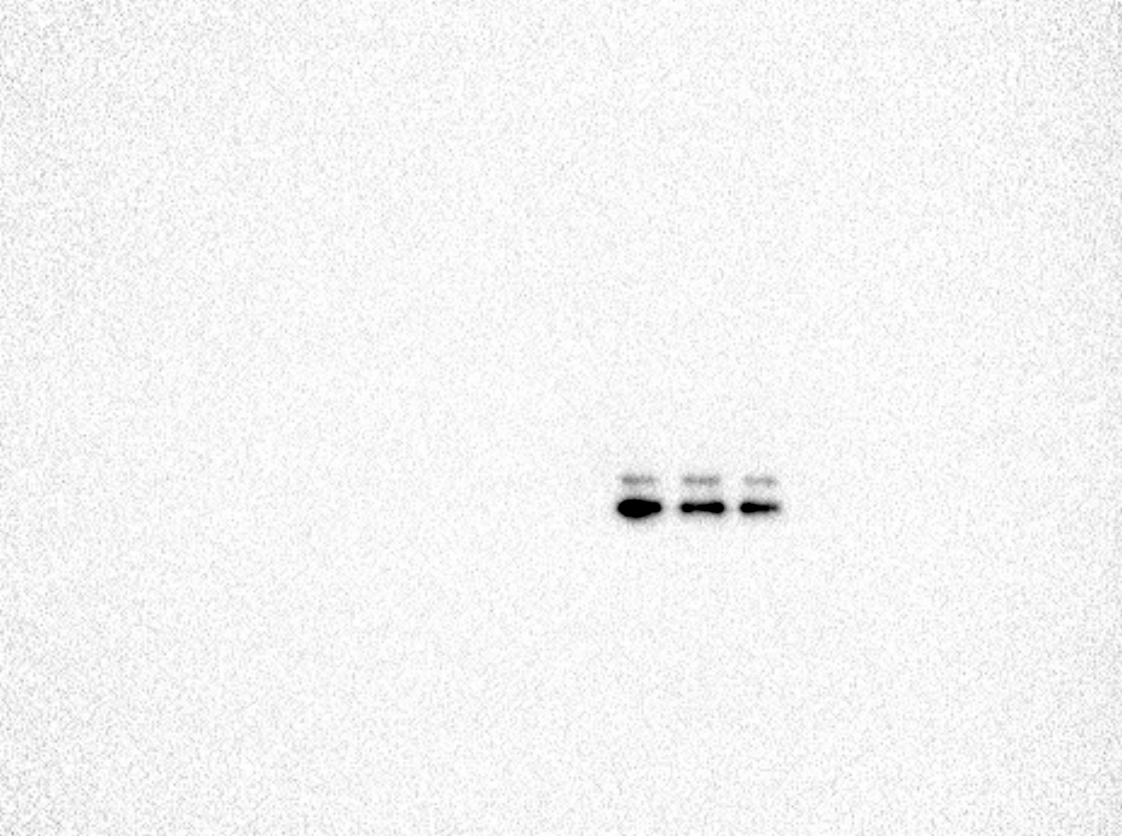

Supplement: S1 File — (ZIP) [file pone.0293530.s003.zip › Uncropped western blots/WB-Cytc/Administrator 2020-08-13 15 ╩▒ 01 ╖╓_Exposure_3.5sec.scn3.tif]

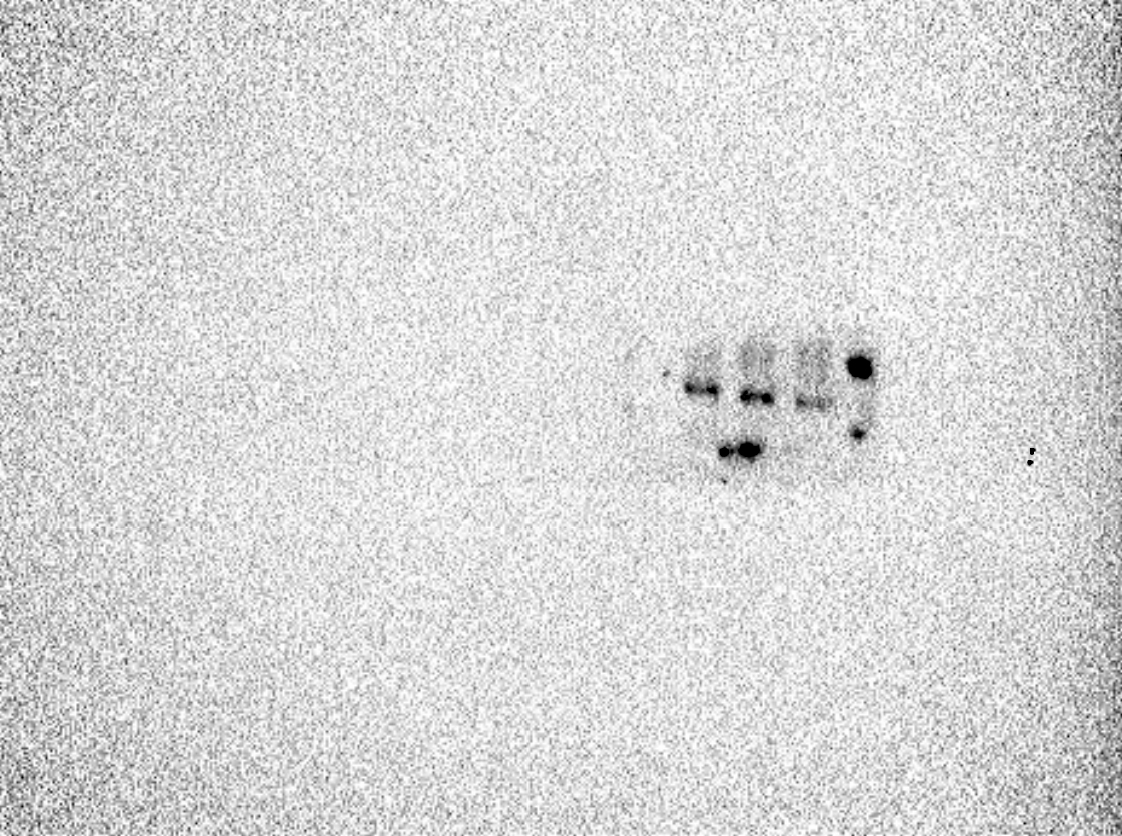

Supplement: S1 File — (ZIP) [file pone.0293530.s003.zip › Uncropped western blots/WB-Cytc/Administrator 2020-08-20 15 ╩▒ 46 ╖╓_Exposure_9.9sec.scn3.tif]

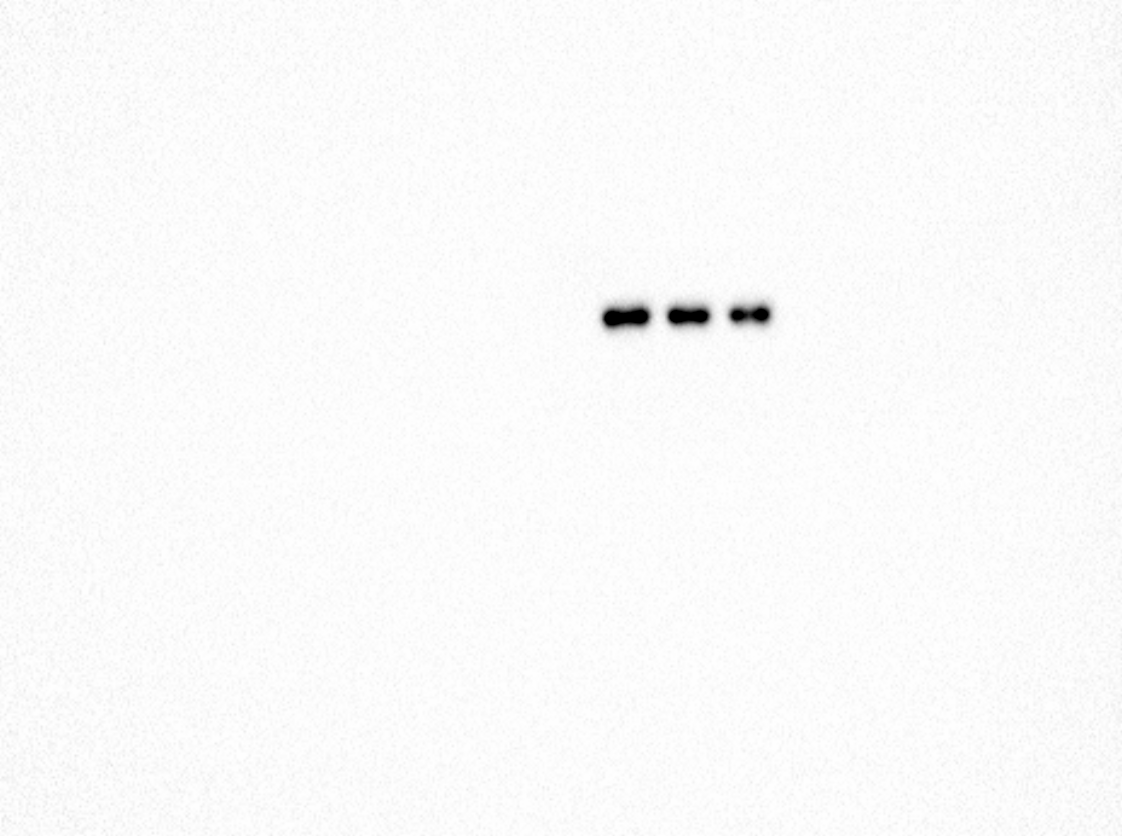

Supplement: S1 File — (ZIP) [file pone.0293530.s003.zip › Uncropped western blots/WB-GAPDH/Administrator 2020-08-07 10 ╩▒ 16 ╖╓_Exposure_2.5sec.scn34.tif]

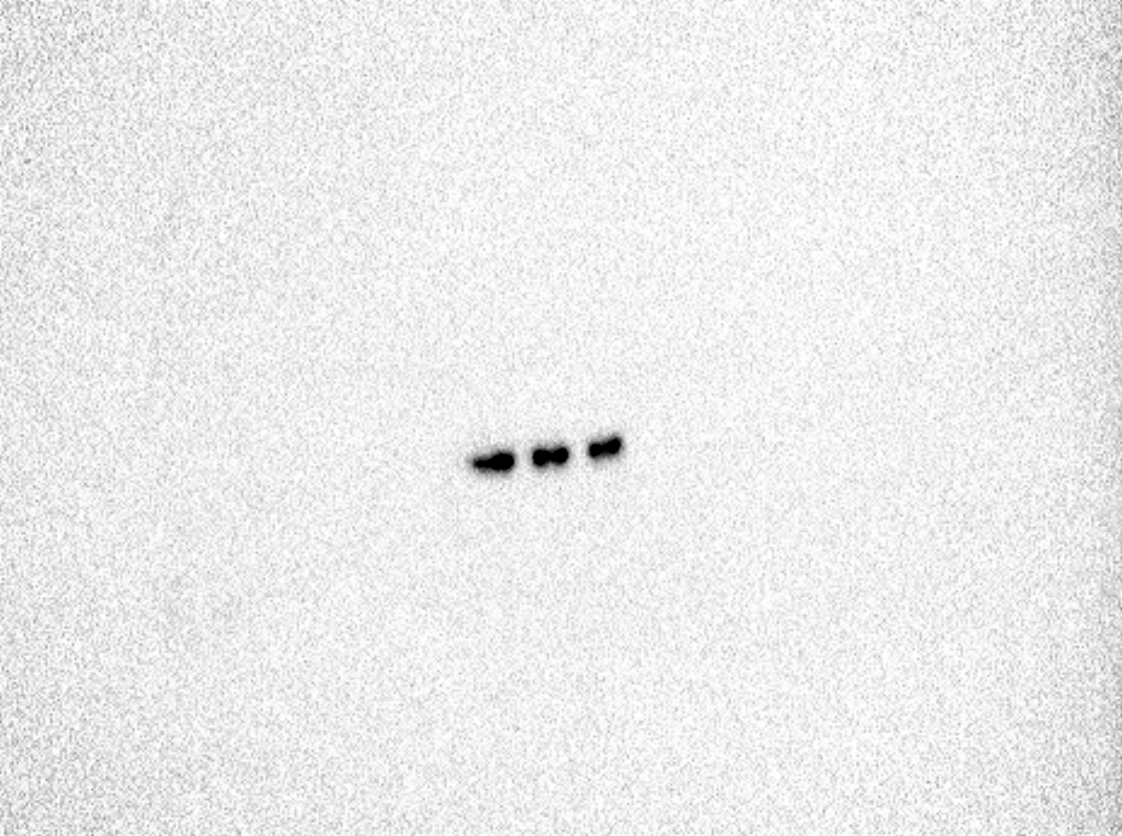

Supplement: S1 File — (ZIP) [file pone.0293530.s003.zip › Uncropped western blots/WB-GAPDH/Administrator 2020-08-12 15 ╩▒ 02 ╖╓_Exposure_9.9sec.scn48.tif]

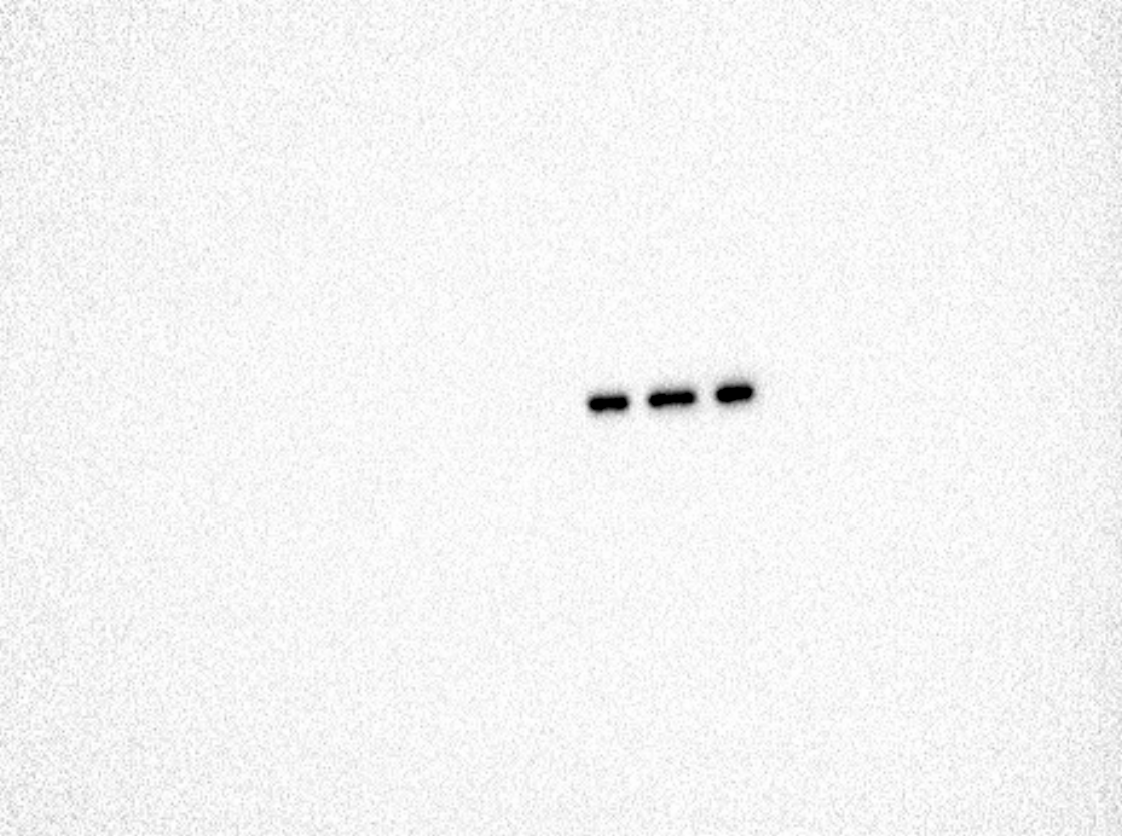

Supplement: S1 File — (ZIP) [file pone.0293530.s003.zip › Uncropped western blots/WB-GAPDH/Administrator 2020-08-18 15 ╩▒ 30 ╖╓_Exposure_5.0sec.scn 10.tif]

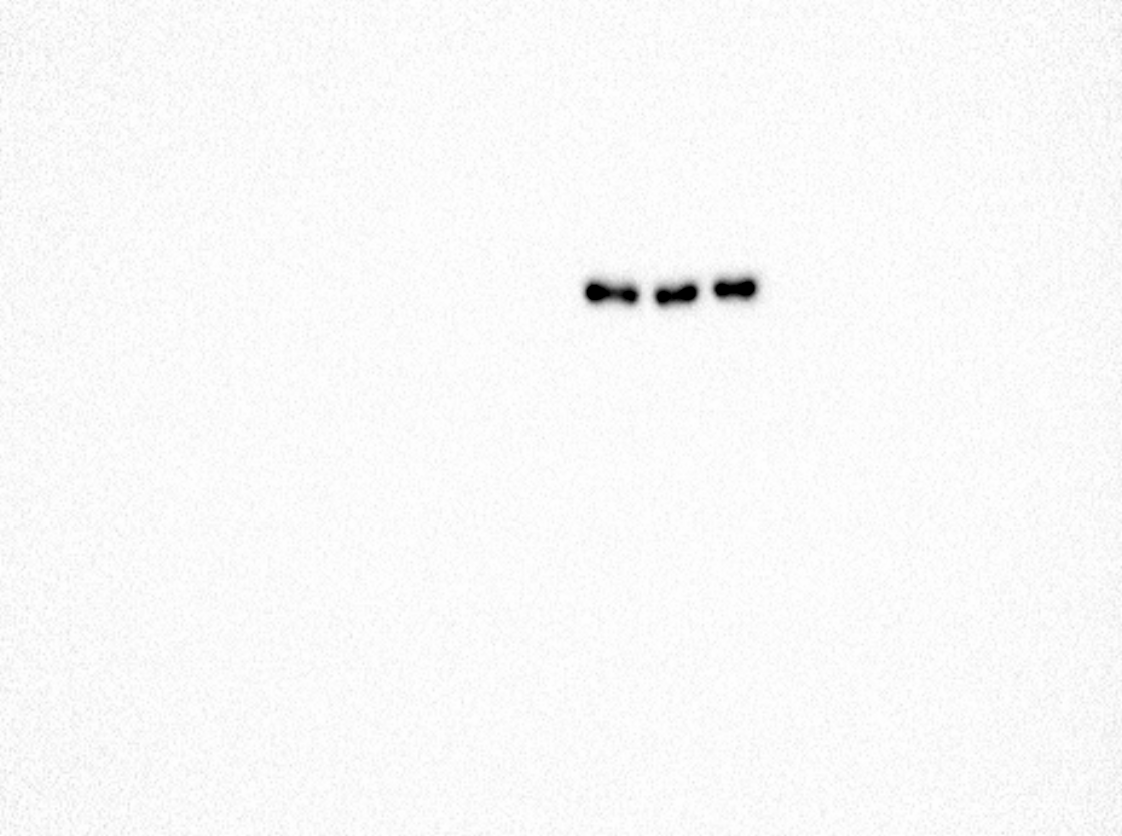

Supplement: S1 File — (ZIP) [file pone.0293530.s003.zip › Uncropped western blots/WB-GAPDH/Administrator 2020-08-20 15 ╩▒ 25 ╖╓_Exposure_3.0sec.scn2.tif]

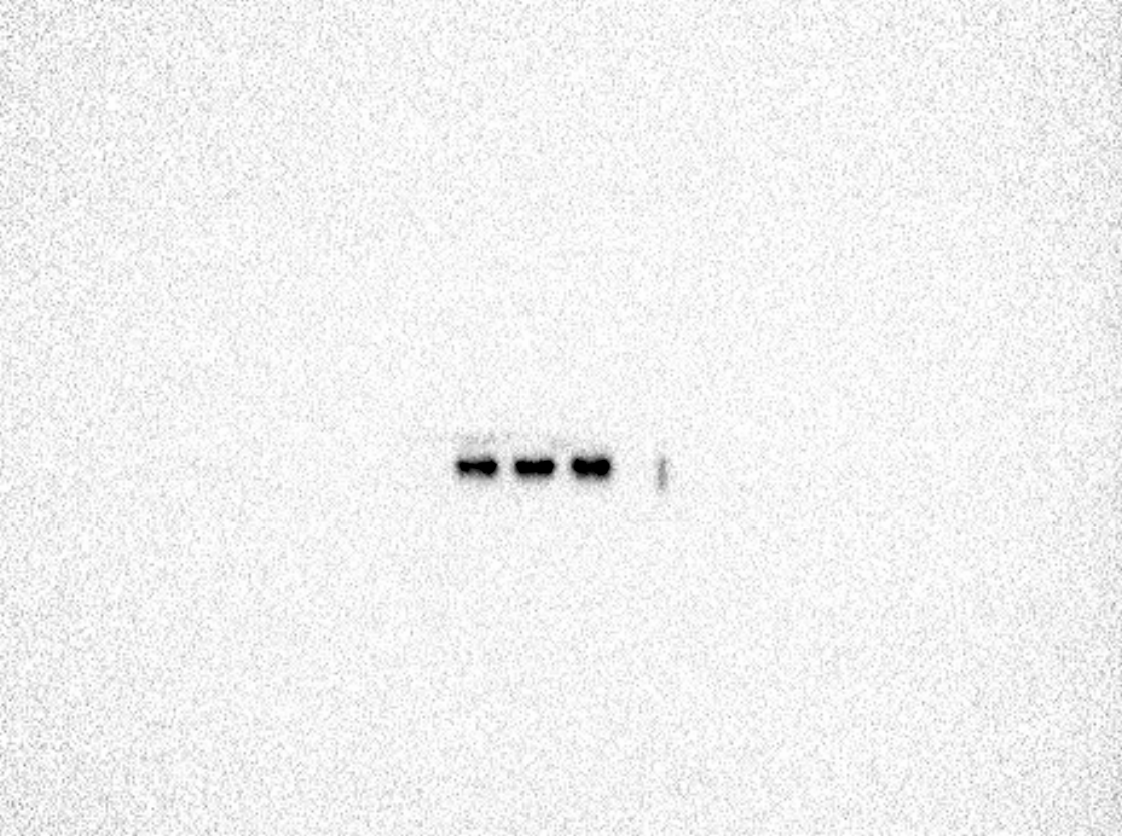

Supplement: S1 File — (ZIP) [file pone.0293530.s003.zip › Uncropped western blots/WB-GAPDH/Administrator 2020-08-21 17 ╩▒ 24 ╖╓_Exposure_2.5sec.scn 50.tif]

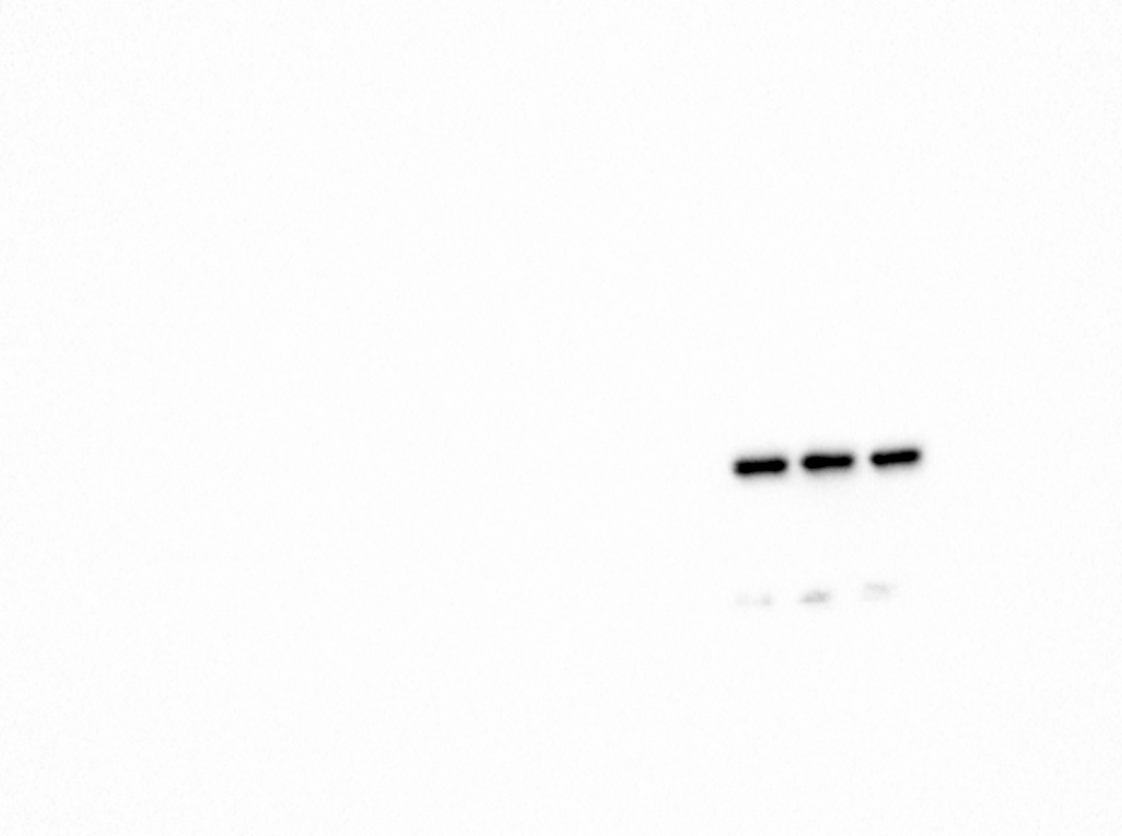

Supplement: S1 File — (ZIP) [file pone.0293530.s003.zip › Uncropped western blots/WB-GAPDH/Administrator 2020-08-25 14 ╩▒ 54 ╖╓_Exposure_1.0sec.scn21.tif]

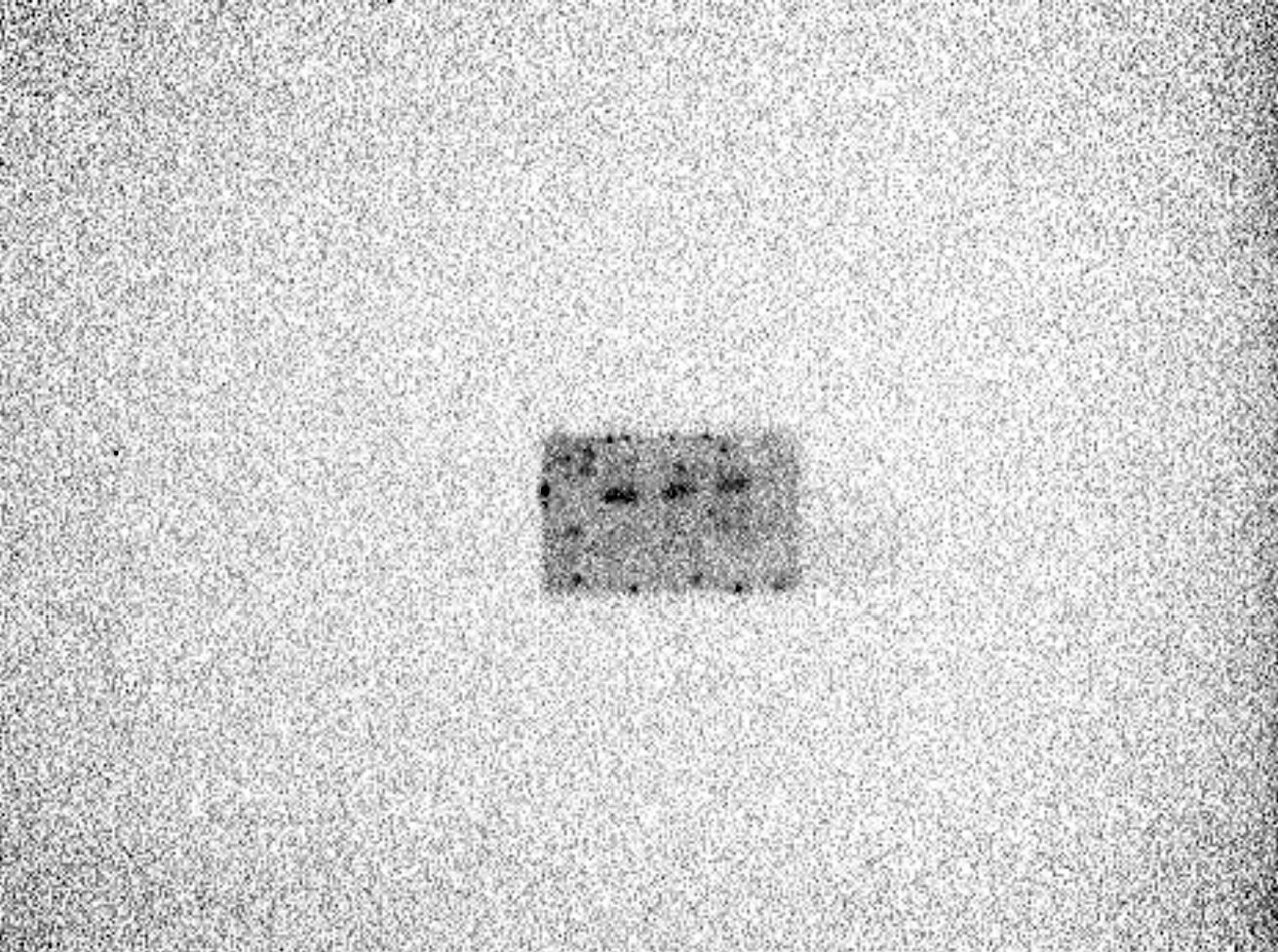

Supplement: S1 File — (ZIP) [file pone.0293530.s003.zip › Uncropped western blots/WB-P53/Administrator 2020-08-04 16 ╩▒ 23 ╖╓_Exposure_33.2sec.tif]

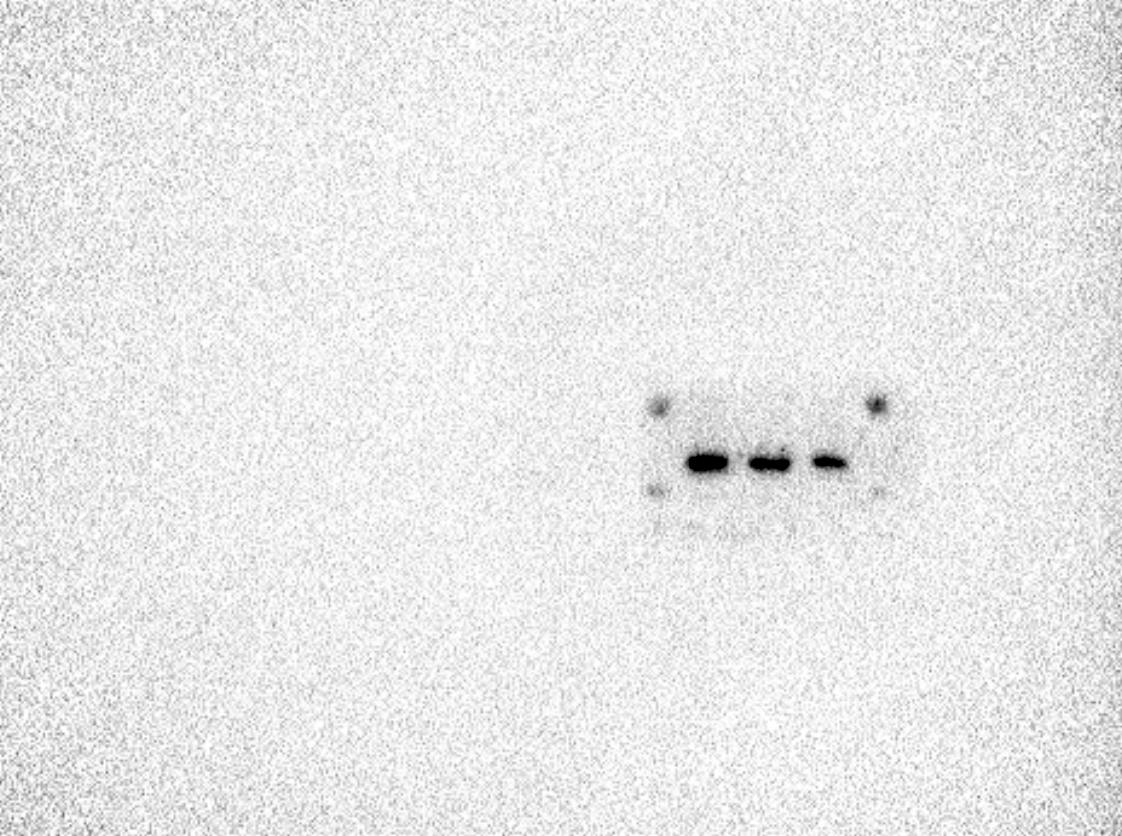

Supplement: S1 File — (ZIP) [file pone.0293530.s003.zip › Uncropped western blots/WB-P53/Administrator 2020-08-15 16 ╩▒ 40 ╖╓_Exposure_10.0sec.tif]

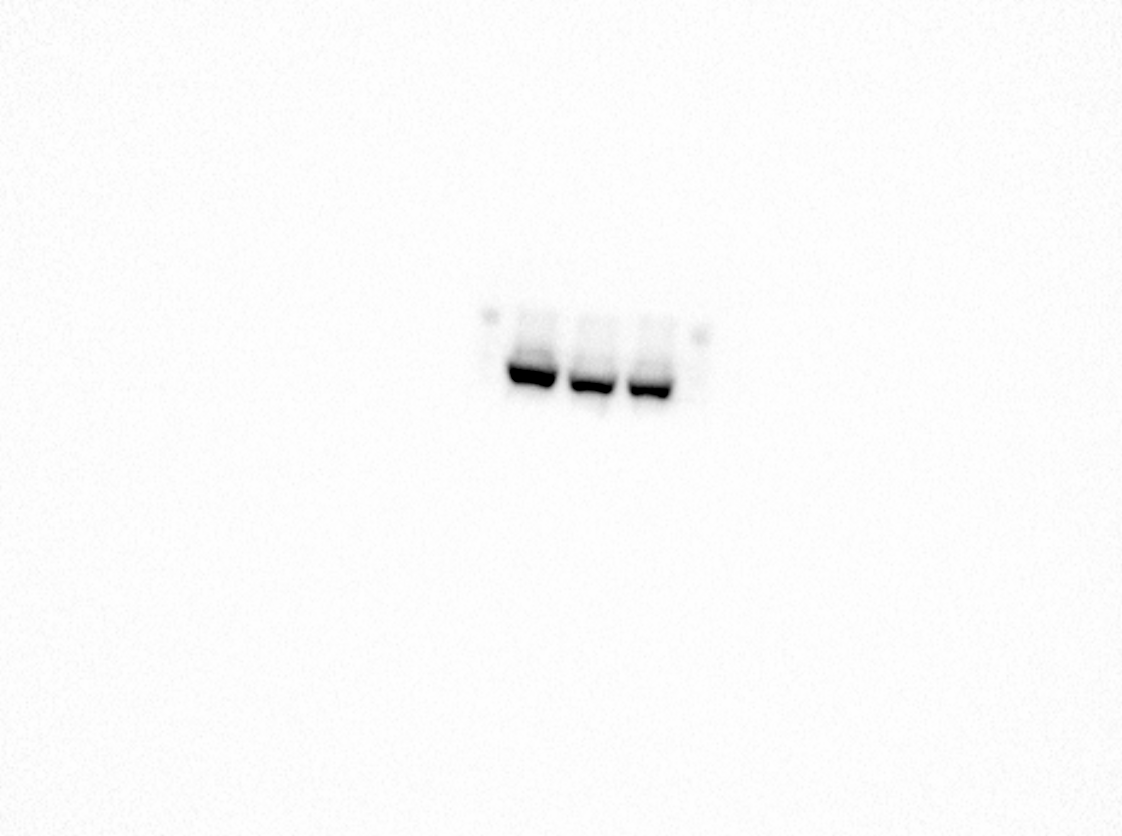

Supplement: S1 File — (ZIP) [file pone.0293530.s003.zip › Uncropped western blots/WB-P53/Administrator 2020-08-21 16 ╩▒ 44 ╖╓_Exposure_1.5sec.scn58.tif]

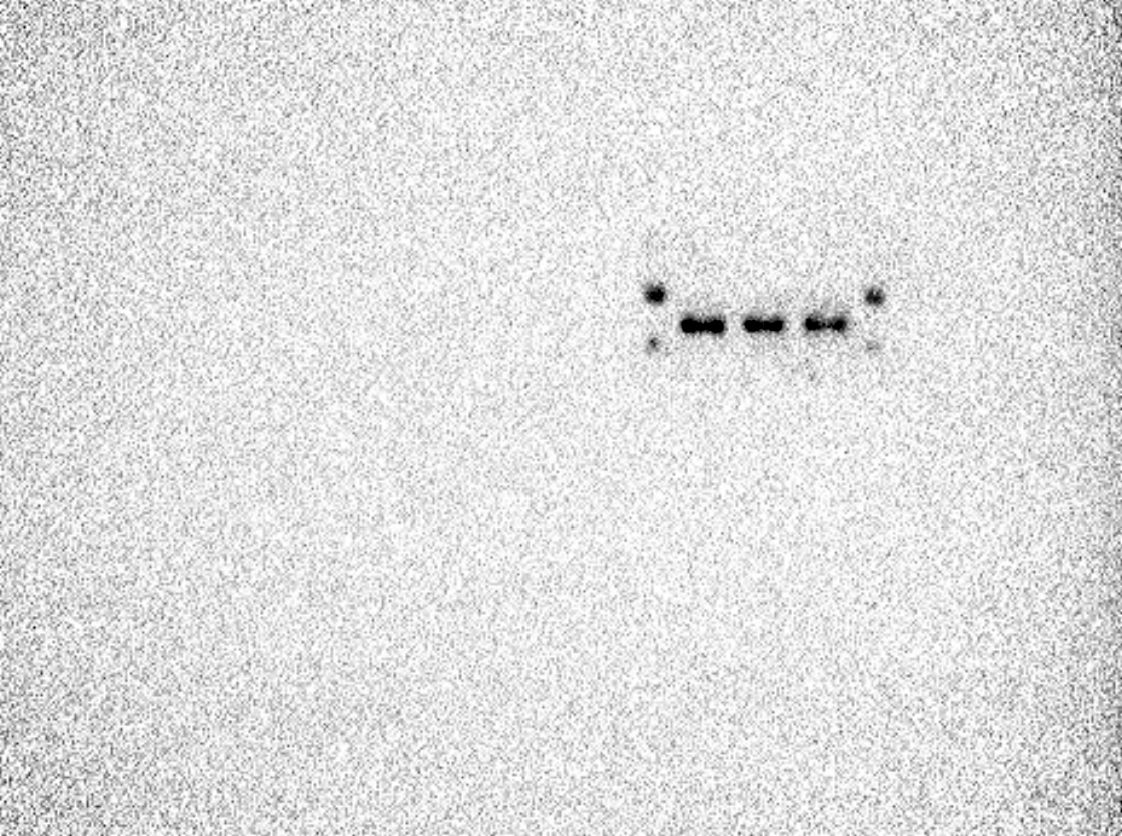

Supplement: S1 File — (ZIP) [file pone.0293530.s003.zip › Uncropped western blots/WB-P53/Administrator 2020-08-21 17 ╩▒ 08 ╖╓_Exposure_5.4sec.scn 55.tif]

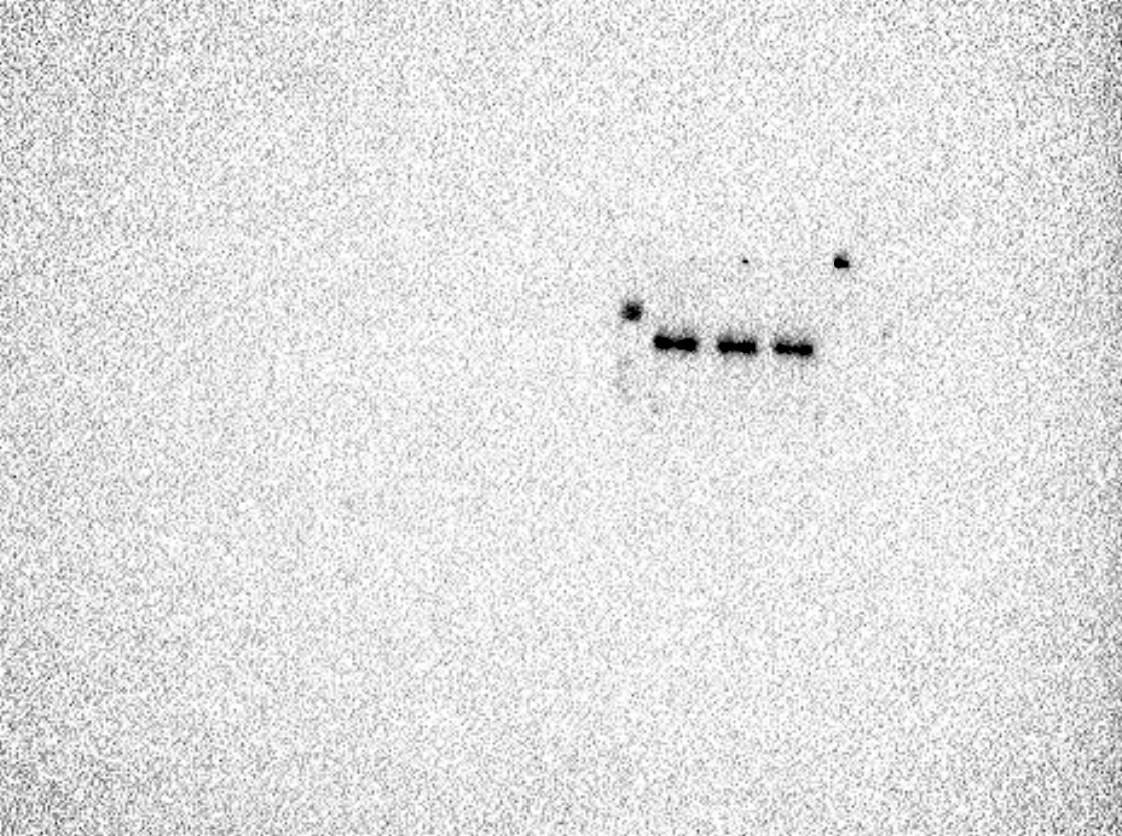

Supplement: S1 File — (ZIP) [file pone.0293530.s003.zip › Uncropped western blots/WB-P53/Administrator 2020-08-21 17 ╩▒ 09 ╖╓_Exposure_5.0sec.scn 56.tif]

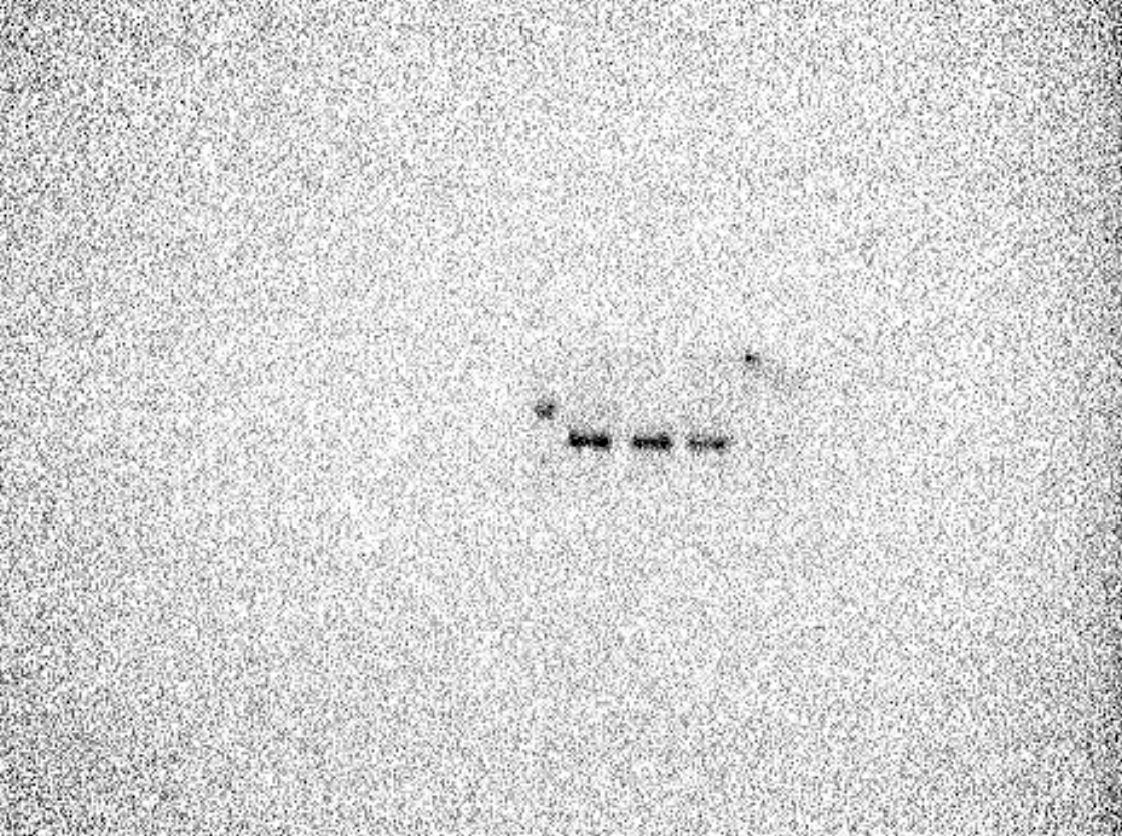

Supplement: S1 File — (ZIP) [file pone.0293530.s003.zip › Uncropped western blots/WB-P53/Administrator 2020-08-21 17 ╩▒ 38 ╖╓_Exposure_4.5sec.scn 56.tif]

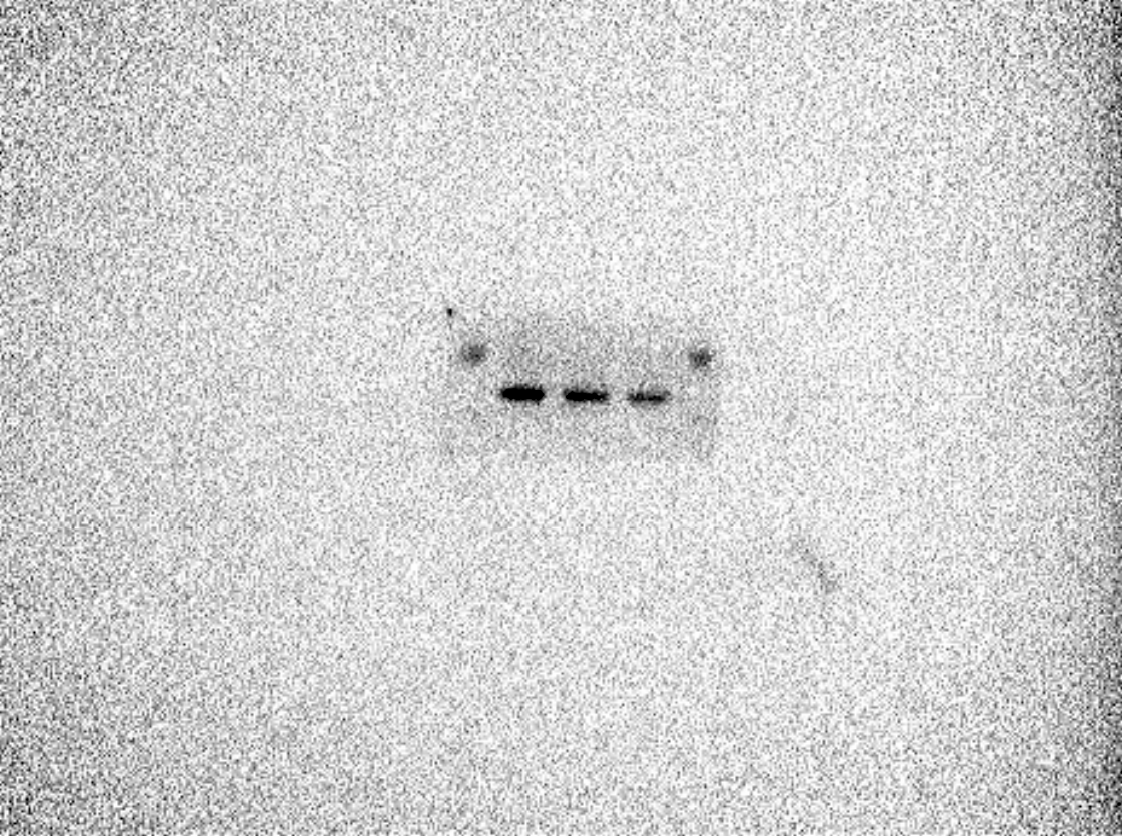

Supplement: S1 File — (ZIP) [file pone.0293530.s003.zip › Uncropped western blots/WB-P53/Administrator 2020-08-22 16 ╩▒ 21 ╖╓_Exposure_11.9sec.tif]

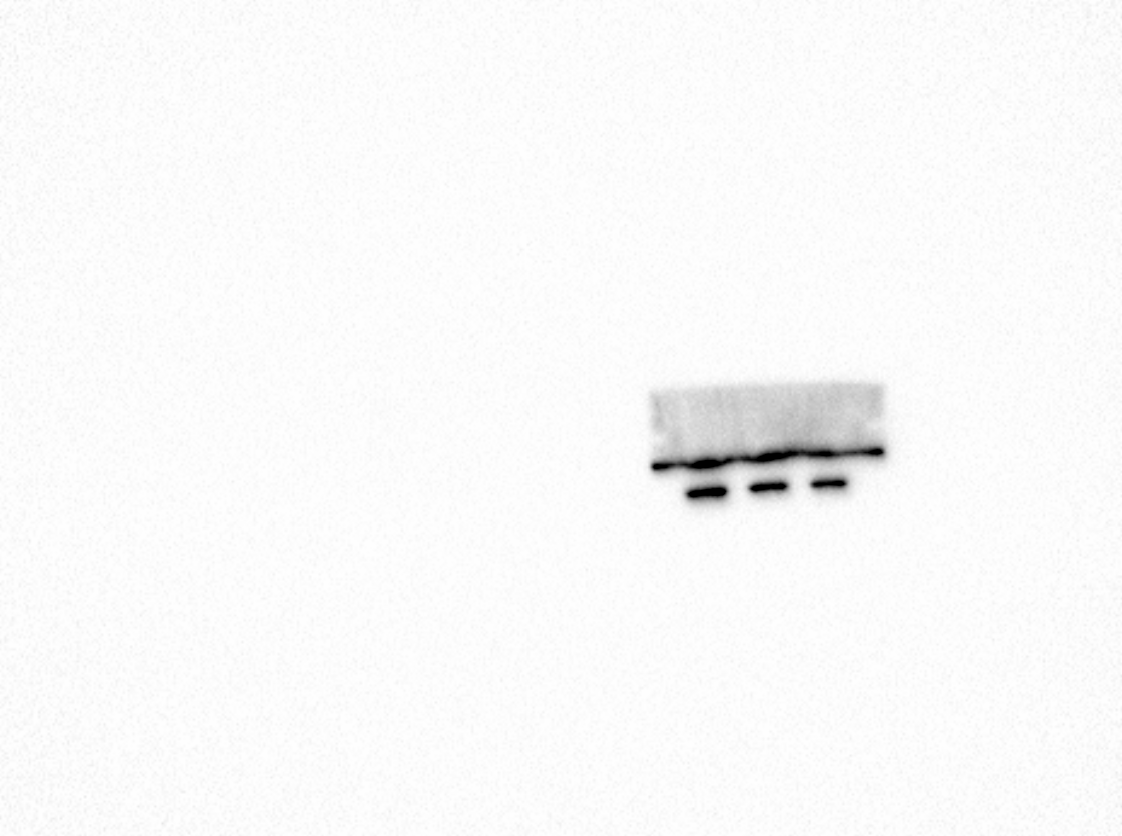

Supplement: S1 File — (ZIP) [file pone.0293530.s003.zip › Uncropped western blots/WB-p85/Administrator 2020-08-07 15 ╩▒ 27 ╖╓_Exposure_1.5sec.scn40.tif]

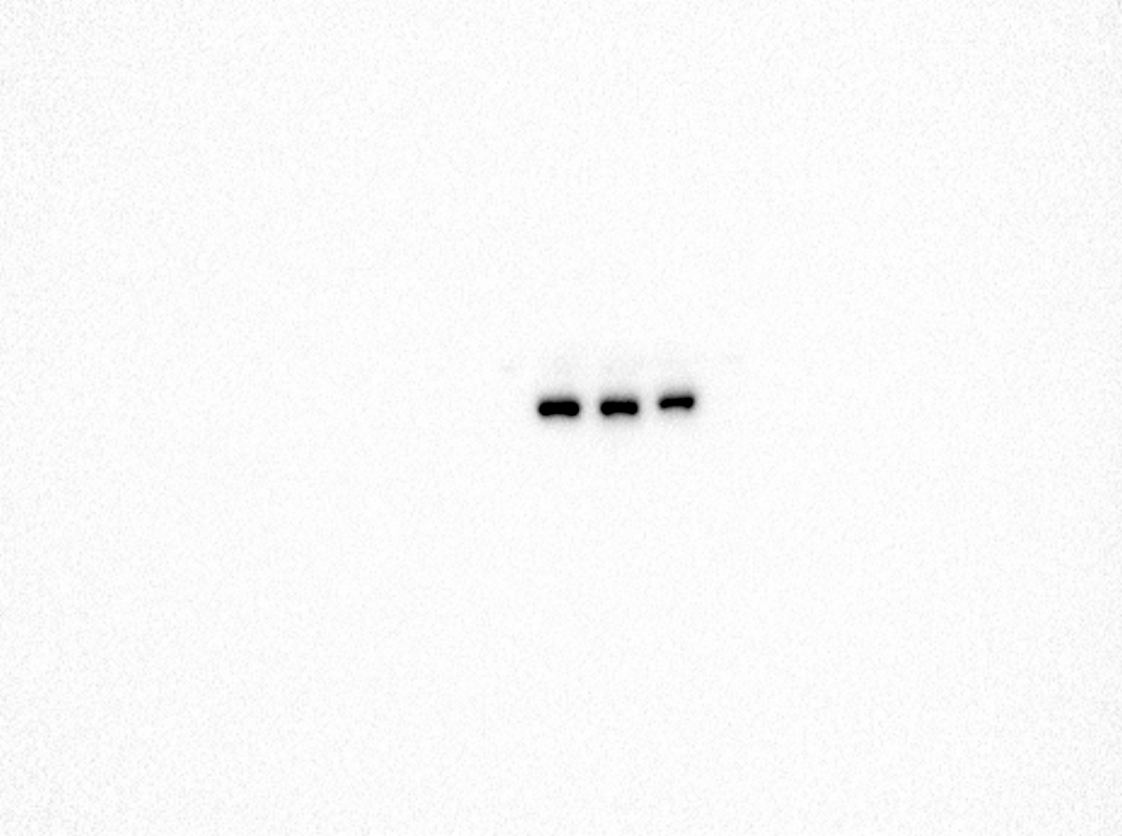

Supplement: S1 File — (ZIP) [file pone.0293530.s003.zip › Uncropped western blots/WB-p85/Administrator 2020-08-12 16 ╩▒ 37 ╖╓_Exposure_3.0sec.scn52.tif]

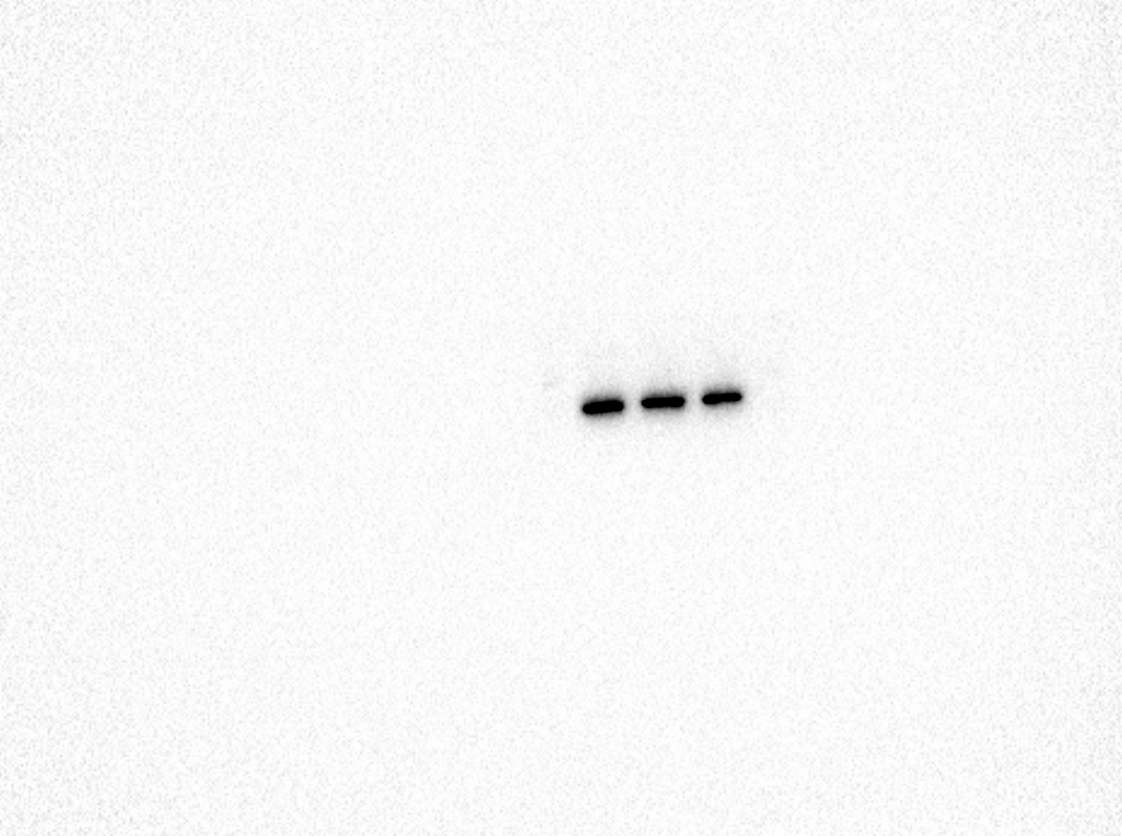

Supplement: S1 File — (ZIP) [file pone.0293530.s003.zip › Uncropped western blots/WB-p85/Administrator 2020-08-14 15 ╩▒ 58 ╖╓_Exposure_2.0sec.scn41.tif]

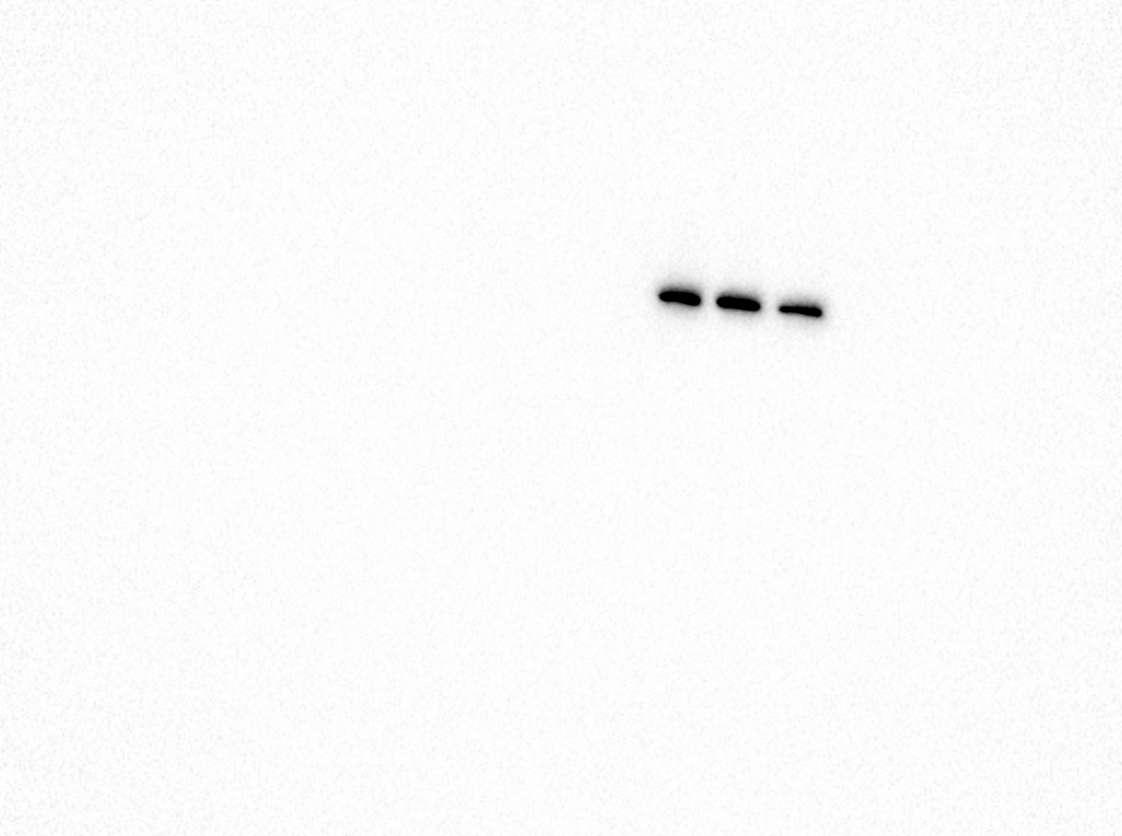

Supplement: S1 File — (ZIP) [file pone.0293530.s003.zip › Uncropped western blots/WB-p85/Administrator 2020-08-18 16 ╩▒ 20 ╖╓_Exposure_1.0sec.scn 21.tif]

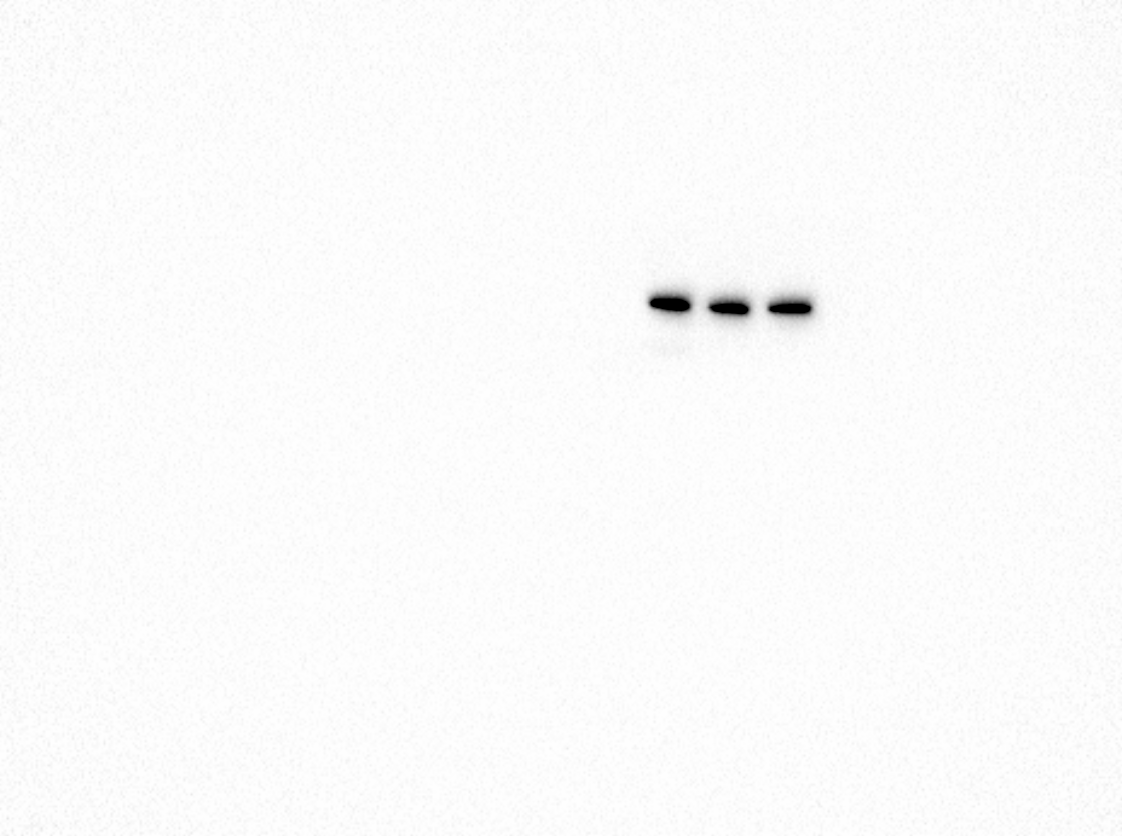

Supplement: S1 File — (ZIP) [file pone.0293530.s003.zip › Uncropped western blots/WB-p85/Administrator 2020-08-18 16 ╩▒ 21 ╖╓_Exposure_1.0sec.scn 15.tif]

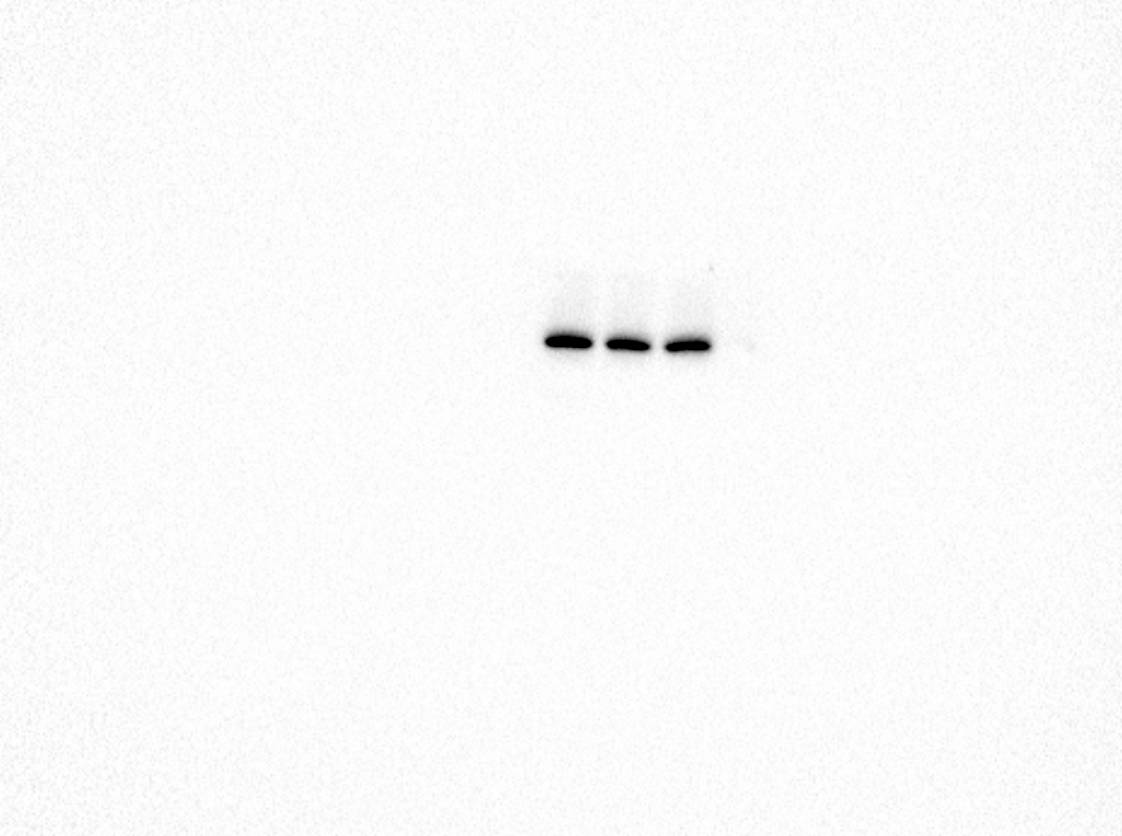

Supplement: S1 File — (ZIP) [file pone.0293530.s003.zip › Uncropped western blots/WB-p85/Administrator 2020-08-22 16 ╩▒ 41 ╖╓_Exposure_1.5sec.tif]
