# Supplementary material for: Cyasterone has a protective effect on steroid-induced Osteonecrosis of the femoral head
Source: PLoS One. 2023 Oct 30;18(10):e0293530. doi: 10.1371/journal.pone.0293530 (PMC10615314; doi:10.1371/journal.pone.0293530)
Supplement: S1 File — (ZIP) [file pone.0293530.s003.zip › Uncropped western blots/Table.docx]

Table 1 Primers of AKT, Bax, P53, P85, BCL-2, Cytochrome C and β-actin R

| **The primers** | **Primer sequences** | **length（nt）** | **The length of the product（bp）** | **The annealing temperature（℃）** |
| --- | --- | --- | --- | --- |
| AKT1 F | TAGGCATCCCTTCCTTACAGC | 21 | 114 | 60.52 |
| AKT1 R | CGCTCACGAGACAGGTGGA | 19 |  |  |
| Bax F | TGGCGATGAACTGGACAACA | 20 | 125 | 60.48 |
| Bax R | CCCAGTTGAAGTTGCCGTCT | 20 |  |  |
| P53 F | ACAGTTAGGGGGTACCTGGC | 20 | 118 | 60.78 |
| P53 R | AGCTCGATGCTCATATCCGAC | 21 |  |  |
| P85 F | ACAAAGCCGAGAACCTATTGC | 21 | 108 | 59.64 |
| P85 R | TGACTTCGCCATCTACCACTAC | 22 |  |  |
| BCL-2 F | GGACGCGAAGTGCTATTGGT | 20 | 141 | 60.52 |
| BCL-2 R | AGTATCCCACTCGTAGCCCC | 20 |  |  |
| Cytochrome C F | CTTGGGCTAGAGAGCGGGA | 19 | 132 | 61.45 |
| Cytochrome C R | GTGGCACTGGGCACACTTTT | 20 |  |  |
| β-actin F | GCCATGTACGTAGCCATCCA | 20 | 375 | 59.5 |
| β-actin R | GAACCGCTCATTGCCGATAG | 20 |  |  |

Note: The primers were designed from the pubmed website https://www.ncbi.nlm.nih.gov/nuccore/?term= and were synthesized by the company of General Biosystems (Anhui) Co., Ltd.
